# Spatiotemporal analysis of mycolactone distribution *in vivo* reveals partial diffusion in the central nervous system

**DOI:** 10.1371/journal.pntd.0008878

**Published:** 2020-12-02

**Authors:** Emma Colucci-Guyon, Aline Rifflet, Sarah Saint-Auret, Anaëlle da Costa, Laurent Boucontet, Thomas Laval, Christophe Prehaud, Nicolas Blanchard, Jean-Pierre Levraud, Ivo G. Boneca, Caroline Demangel, Laure Guenin-Macé

**Affiliations:** 1 Macrophages and Development of Immunity, Institut Pasteur, CNRS UMR 3738, Paris, France; 2 Institut Pasteur, Unité Biologie et génétique de la paroi bactérienne, Paris 75724, France; CNRS, UMR 2001 “Microbiologie intégrative et moléculaire”, Paris 75015, France; INSERM, groupe Avenir, Paris, France; 3 Université de Haute-Alsace, Université de Strasbourg, CNRS, LIMA, UMR 7042, Mulhouse, France; 4 Viral Neuroimmunology, Institut Pasteur, Paris, France; 5 Immunobiology of Infection Unit, Institut Pasteur, INSERM U1221, Paris, France; 6 Université Paris Diderot, Sorbonne Paris Cité, Paris, France; Johns Hopkins University, UNITED STATES

## Abstract

*Mycobacterium ulcerans*, the causative agent of Buruli ulcer (BU) disease, is unique amongst human pathogens in its capacity to produce a lipid toxin called mycolactone. While previous studies have demonstrated that bacterially-released mycolactone diffuses beyond infection foci, the spatiotemporal distribution of mycolactone remained largely unknown. Here, we used the zebrafish model to provide the first global kinetic analysis of mycolactone’s diffusion *in vivo*, and multicellular co-culture systems to address the critical question of the toxin’s access to the brain.

Zebrafish larvae were injected with a fluorescent-derivative of mycolactone to visualize the *in vivo* diffusion of the toxin from the peripheral circulation. A rapid, body-wide distribution of mycolactone was observed, with selective accumulation in tissues near the injection site and brain, together with an important excretion through the gastro-intestinal tract. Our conclusion that mycolactone reached the central nervous system was reinforced by an *in cellulo* model of human blood brain barrier and a mouse model of *M*. *ulcerans*-infection.

Here we show that mycolactone has a broad but heterogenous profile of distribution *in vivo*. Our investigations *in vitro* and *in vivo* support the view that a fraction of bacterially-produced mycolactone gains access to the central nervous system. The relative persistence of mycolactone in the bloodstream suggests that assays of circulating mycolactone are relevant for BU disease monitoring and treatment optimization.

## Author summary

Mycolactone is the major virulence factor of *Mycobacterium ulcerans*, the human pathogen causing Buruli ulcer (BU) disease. While it is now established that mycolactone is able to diffuse from infected tissues to exert immunomodulatory and analgesic effects at the systemic level, the *in vivo* spatiotemporal distribution of mycolactone remained largely unknown. Here, using the zebrafish larva, we describe the spatiotemporal distribution of a fluorescent derivative of mycolactone *in vivo*. We show that fluorescent mycolactone quickly diffuses from the blood circulation into various organs, and accumulates in muscles and brain. Mycolactone’s diffusion into the central nervous system was further confirmed in the mouse model of *M*. *ulcerans* infection. We reported previously that mycolactone suppresses the production of inflammatory mediators by primary microglia at nanomolar concentrations *in vitro* [1]. In the present study, we provide evidence suggesting that bacterially-produced mycolactone is able to reach the brain. While additional *in vivo* investigations will be required, we can speculate that the amount of mycolactone reaching the brain in the context of *M*. *ulcerans* infection is sufficient to modulate inflammation and pain transmission.

## Introduction

Macrolidic polyketide Mycolactone (ML) is the major virulence factor of *Mycobacterium ulcerans*, the human pathogen causing Buruli ulcer (BU) disease [2–4]. BU manifests as necrotic cutaneous lesions around bacterial foci, with a distinctive lack of inflammation and pain [5]. While bacteria remain localized at the site of infection in the skin, ML diffuses from infected tissues to exert immunomodulatory and analgesic effects at the systemic level [1,6]. In patients with active BU, liquid chromatography tandem mass spectrometry (LC-MS/MS) has identified structurally intact ML in serum [7]. In mice experimentally infected with *M*. *ulcerans*, the distinctive mass spectrometric signature of ML was detected in blood cells, spleen, liver and kidneys [8]. While these observations suggest that ML diffuses broadly into infected organisms, the spatiotemporal distribution of ML *in vivo* remained largely unknown.

Recent studies have shown that ML mediates its cytopathic effects by targeting the host Sec61 translocon [9,10]. ML binding to human Sec61 prevents the translocation of newly synthetized membrane and secreted proteins into the endoplasmic reticulum, leading to their cytosolic degradation [10,11]. In immune cells, an immediate effect of this molecular blockade is a decreased ability to produce cytokines and cytokine receptors that are essential to mount inflammatory responses [12,13]. Sustained Sec61 blockade triggers pro-apoptotic stress responses, providing an explanation for how bacterially-produced ML causes host cell death at the infection site [14]. Notably, we found recently that ML treatment impairs the activation-induced inflammatory responses and long-term viability of sensory neurons, Schwann cells and microglia in a Sec61-dependent manner [1]. In intrathecally-injected rats, ML decreased significantly the basal level of inflammatory cytokines in the spinal cord [1]. Therefore, the anti-inflammatory effect of ML on the nervous system could also contribute to analgesia, a distinctive hallmark of BU. However, whether ML is able to cross the blood-brain barrier (BBB) and gain access to the cells of the central nervous system is currently unknown.

Originally introduced as a model organism in developmental biology, the zebrafish (*Danio rerio*) has emerged in recent years as a powerful vertebrate model to study host-pathogen interactions, human diseases, drug screening and toxicology due to its >70% genetic homology with humans and remarkable conservation of biological pathways, major organs and tissues [15–18]. ML-producing bacteria cause BU-like disease in infected fish [19] and cytotoxicity in cultured fish macrophages [20], suggesting that fish Sec61 is susceptible to inhibition by ML. In support of this hypothesis, Sec61α, the central subunit of the human translocon that is targeted by ML, is conserved at 97% in *Danio rerio* (S1 Fig). Due to their optical transparency and small size, zebrafish larvae allow the non-invasive tracking of fluorescent compounds throughout the entire body *in vivo* at high resolution. Moreover, the development, structure and function of the BBB being conserved between mammals and zebrafish, this model provides a means to test the permeability of the BBB to ML [21–23]. Here, we used a fluorescent surrogate of ML [24–26] and a combination of *in vitro* and *in vivo* approaches to investigate the spatiotemporal distribution of ML at the whole-organism scale and address the critical question of its access to the brain.

## Methods

### Ethics statement

All experiments on zebrafish were done on larvae < 5 dpf, in consequence they are not submitted to authorization. Mouse experiments were approved by the committee on animal experimentation of the Institut Pasteur and by the French Ministry of Research. Authorization #11141.

### Mycolactone and fluorescent derivatives

ML was purified from the Malaysian human isolate *M*. *ulcerans* 1615, then quantified by spectrophotometry (λmax = 362 nm; log ε = 4.29) [27]. Stock solutions were prepared in ethanol then diluted 2x in PBS in glass vials for zebrafish experiments or in DMSO then diluted 750x in culture medium for cellular assays. In all cases, controls corresponding to the same volume of vehicle were included. An optimized synthesis of Bodipy-mycolactone (Bdpy-ML) and saturated Bodipy-mycolactone (Sat-Bdpy-ML) is described in S1 file.

### Zebrafish care and maintenance

Zebrafish transgenic lines Tg(*Kdrl*:*HsHRAS-mCherry*) (s916Tg) also referred to as Tg(*kdrl*:*Ras-mCherry*) [28] and Tg*(mfap4*:*mCherry-F)* (ump6Tg) [29] were raised in our facility and maintained according to standard procedures [30]. Briefly, embryos were obtained by natural spawning, bleached according to standard protocols, and then kept in Petri dishes containing Volvic source water and, from 24 hours post fertilization onwards 0.003% 1-phenyl-2-thiourea (PTU) (Sigma-Aldrich) was added to prevent pigmentation. Embryos were reared at 28°C or 24°C according to the desired speed of development; injected larvae were always kept at 28°C. All timings in the text refer to the developmental stage at the reference temperature of 28.5°C. Larvae were injected at 4 days post-fertilization (dpf), stage at which the BBB is functional [23]. Larvae were anesthetized with 200μg/ml tricaine (Sigma-Aldrich) during the injection procedure as well as during *in vivo* imaging and euthanized with 1mg/ml tricaine before processing for whole-mount immunohistochemistry and TUNEL staining.

### Microinjections of zebrafish larvae

Zebrafish larvae (4 dpf) were anaesthetized by immersion in buffered tricaine (Sigma). They were injected in the caudal vein (bloodstream injection) with 1.5 nl of Bdpy- or Sat-Bdpy-ML solutions concentrated at 7.5 mg/ml in ethanol in glass vial then diluted in PBS (1:1), using pulled borosilicate glass microcapillary (GC100F-15 Harvard Apparatus) pipettes under a stereomicroscope (Stemi 2000, Carl Zeiss, Germany) with a mechanical micromanipulator (M-152; Narishige), and a Picospritzer III pneumatic microinjector (Parker Hannifin) set at a pressure of 20 p.s.i. and an injection time of 40 ms as previously described [31,32]. For TUNEL assays, larvae were injected with 0.25, 0.5 or 1ng ML in a final volume of 1.5 nl ethanol: PBS (1:1, v/v). Injected larvae were transferred into individual wells (containing 1ml of Volvic water + 0.003% PTU in 24-well culture plates) and incubated at 28°C until 24 hours post injection (hpi).

### Confocal fluorescence imaging

High resolution confocal live imaging of injected larvae was performed as previously described [33]. Briefly, injected larvae were positioned in lateral or dorsal position in 35 mm glass-bottom μ-dishes (Ibidi Cat#: 81158). Larvae were immobilized in the dish using a 1% low-melting-point agarose solution (Promega; Cat#: V2111) then covered with Volvic water containing tricaine. A Leica SP8 confocal microscope equipped with two PMT and Hybrid detector, a 20X IMM objective (HC PL APO CS2 20X/0.75), a X–Y motorized stage and the LAS-X software were used to live image injected larvae. To generate images of the whole larvae, a mosaic of confocal z-stack images was taken with the 20X objective using the Tile Scan tool of the LAS-X software and was stitched together using the Mosaic Merge tool of the LAS-X software. All samples were acquired using the same settings, allowing comparisons of independent experiments. After acquisitions, larvae were washed and transferred in a new 24-well plate filled with 1 ml of fresh water in each well, incubated at 28°C and imaged again under the same conditions the day after.

### Whole-mount immunohistochemistry and TUNEL staining

For the detection of apoptosis, euthanized zebrafish larvae were fixed in 4% methanol-free formaldehyde overnight then kept in methanol at −20°C for at least 2h. TUNEL staining was performed using ApopTag Red (Sigma-Aldrich #S7165) combined with peroxidase (POD) coupled anti-DIG antibody (Roche #11207733910, 1:200) followed by tyramide-based amplification as previously described [34]. To simultaneously visualize the transgenic reporter markers of macrophages, rabbit anti-DsRed (Clontech #632496, 1:300) antibody was co-incubated with the POD-coupled anti-DIG antibody. POD-coupled antibodies were detected by an incubation with 1:100 Cy3-Tyramide (Millipore) in PBS, 0.1 mol/L imidazole, and 0.001% H_2_O_2_ in the dark for 60 minutes, and finally anti-DsRed antibodies were revealed by anti-rabbit-AlexaFluor 633 (Life Technologies; #A21070, 1:200) coupled antibody as previously described [35]. Larvae were progressively embedded in glycerol (for conservation and microscopic observation) and mounted in lateral or dorsal position for confocal fluorescent imaging as described above.

### Image processing and analysis

Raw data generated with the Leica SP8 and LAS-X software were processed and analyzed using the Icy open source platform [36]. For quantification of Bdpy-ML accumulation, all acquisitions were done with the same settings and mean fluorescence intensity was measured in defined region of interest, in at least two different focal planes for each region. Microglia morphology was analyzed using the HK-Mean plugin of the Icy software.

### *In cellulo* model of blood-brain barrier system

The human cerebral microvessels endothelial cells hCMEC/D3 [37] were purchased from Tebu-Bio (France). They were grown at 37°C on rat collagen (0.1 mg/ml in water, Cultrex, 3443-100-01, R&D Systems, U.K.) in EndoGro medium (Merck Millipore, SCME004, France) supplemented with 5% fetal bovine serum (FBS) and 1% penicillin-streptavidin (Thermofisher, 15140, France) as described in [38]. U373 cells (ECACC #08061901) were maintained in DMEM supplemented with 5% FBS and 1% penicillin-streptavidin (Thermofisher, 15140, France). For BBB experiments, 2.10^5^ hCMEC/D3 cells were seeded on collagen-coated cell culture 12-well, 0.4 μm porosity PET transwell inserts (Corning, 3460; Corning, 3450, USA) and grown for 6 days. At day 6, inserts containing hCMEC/D3 cells were placed in wells containing U373 cells seeded in presence of EndoGro medium. For transport experiments, ML or Bdpy-ML were added in the apical compartment respectively at a final concentration of 1μg/ml and 2μg/ml in supplemented EndoGro medium. Incubation was done at 37°C in presence of the insert during 15 min to 6 hours before removal of the insert. Incorporation of Bdpy-ML in hCMEC/D3 and U373 cells was assessed by FACS following detachment with Trypsine-EDTA solution (Sigma). For detection of ML, U373 cells were activated overnight by addition of LPS (1μg/ml). The release of IL-8 in the supernatant was assessed by ELISA using the ELISA MAX standard set human (Biolegend).

### Assessment of the restrictive paracellular permeability with Lucifer yellow

The restrictive paracellular permeability of hCMEC/D3 was assessed by their low permeability to the non-permanent fluorescent Lucifer yellow (LY) (Sigma Aldrich, L0259, USA) as previously described [39]. Briefly, for 12-well inserts, hCMEC/D3 monolayers cultivated for 6–7 days on inserts, were transferred to 12-well plates containing 1.5 mL of transport medium [(HBSS, Thermofisher, 14025–100, France) supplemented by 10 mM of hepes (Thermofisher, 15630–080, France) and 1 mM of sodium pyruvate (Thermofisher, 11360)] per well (abluminal compartment). Transport medium (0.5 mL) containing 50 μM of LY was then added to the luminal compartment. After 10, 25 and 45 min of incubation (37°C, 5% CO2, 95% humidity), the inserts were transferred into new wells containing 1.5 mL of transport medium. Aliquots were taken for each time point, from both compartments and the concentration of LY determined using a fluorescence spectrophotometer (Tecan Infinite F500, USA).

The endothelial permeability coefficient (Pe) of LY was calculated in centimeters/min (cm/min) [40]. To obtain a concentration-independent transport parameter, the clearance principle was used. Briefly, the average volume cleared was plotted versus time, and the slope was estimated by linear regression. Both insert permeability (PSf, for insert only coated with collagen) and insert plus endothelial cell permeability (PSt, for insert with collagen and cells) were taken into consideration, according to the following formula: 1/PSe = 1/PSt − 1/PSf. The permeability value for the endothelial monolayer was then divided by the surface area of the porous membrane of the insert to obtain the endothelial permeability coefficient of the molecule in cm.min−1 (see calculation matrix in S2 File). As a quality control, we considered that a Pe above 1.2 indicated an altered BBB integrity [39]. The integrity of the endothelial layer was controlled at the end of transport experiments with vehicle, Bdpy-ML and ML.

### Mouse infection with Mycobacterium ulcerans

Eight-week-old female mice (C57BL/6JRj) were housed and bred under specific-pathogen-free conditions with food and water *ad libitum*. Mice have been infected with a bioluminescent strain *of M*. *ulcerans* (JKD8049 containing a bioluminescent reporter plasmid pMV306 hsp16+luxG13)[41] allowing us to follow the course of infection by measuring the increase of bioluminescence. 30 μl of bacterial suspension or PBS were inoculated subcutaneously in each rear footpad at an estimated concentration of 2.10^3^ colony forming unit per inoculum in mice anesthetized by intraperitoneal injection of Ketamine/ Xylazine. Mice were imaged weekly using an IVIS spectrum (PerkinElmer) and were sacrificed 11 weeks post-infection, when the bioluminescence reached a plateau.

### LC-MS/MS analysis

Mice were deeply anesthetized intraperitoneally with Ketamine/ Xylazine then perfused with PBS. Organs were collected and frozen on dry ice. Total lipids were extracted overnight from homogenized organs with chloroform-methanol (2:1, vol/vol) at 4°C. The organic phase was collected after addition of 0.2 volumes of deionized water, dried under nitrogen and resuspended in ice-cold acetone. The acetone-soluble fraction was resuspended in ethanol for sampling preservation. Half of the samples were dried by vacuum centrifuge and were resuspended in a mix of water/acetonitrile (50:50, vol/vol) acidified with 0.1% formic acid (FA) for analysis by LC:MS/MS. 10 μl of lipid extracts from pooled organs were injected into the analytical column (Luna Omega PS C18, 1,6 μm 150x2.1 mm, Phenomenex) of a UHPLC Ultimate 3000 Dionex chromatographic system. This device was coupled to a high-resolution Q-Orbitrap (Q-Exactive Focus, ThermoFisher Scientific) mass spectrometer fitted with electrospray ionization source (ESI). ML was eluted by a 20 min gradient from 50% to 95% (Buffer A: H_2_O, 0.1% FA; Buffer B: CH_3_CN, 0.1% FA) at a flow rate of 500 μL/min. Full-scan MS was performed in positive ion mode with a ion spray voltage of 3.5 kV from 120 to 1,200 m/z. MS/MS of ML was performed on [M+Na]+ (m/z 765.4913) at a normalized collision energy of 35%.

### Statistical analyses

Statistical analyses and graphical representations were performed using GraphPad Prism software (8.3.1, La Jolla, CA). Unpaired non-parametric Mann-Whitney tests were used as statistical tests.

## Results

### In zebrafish larva, intravenously-delivered ML has a body-wide distribution and reaches the brain within hours

**Fig 1:**
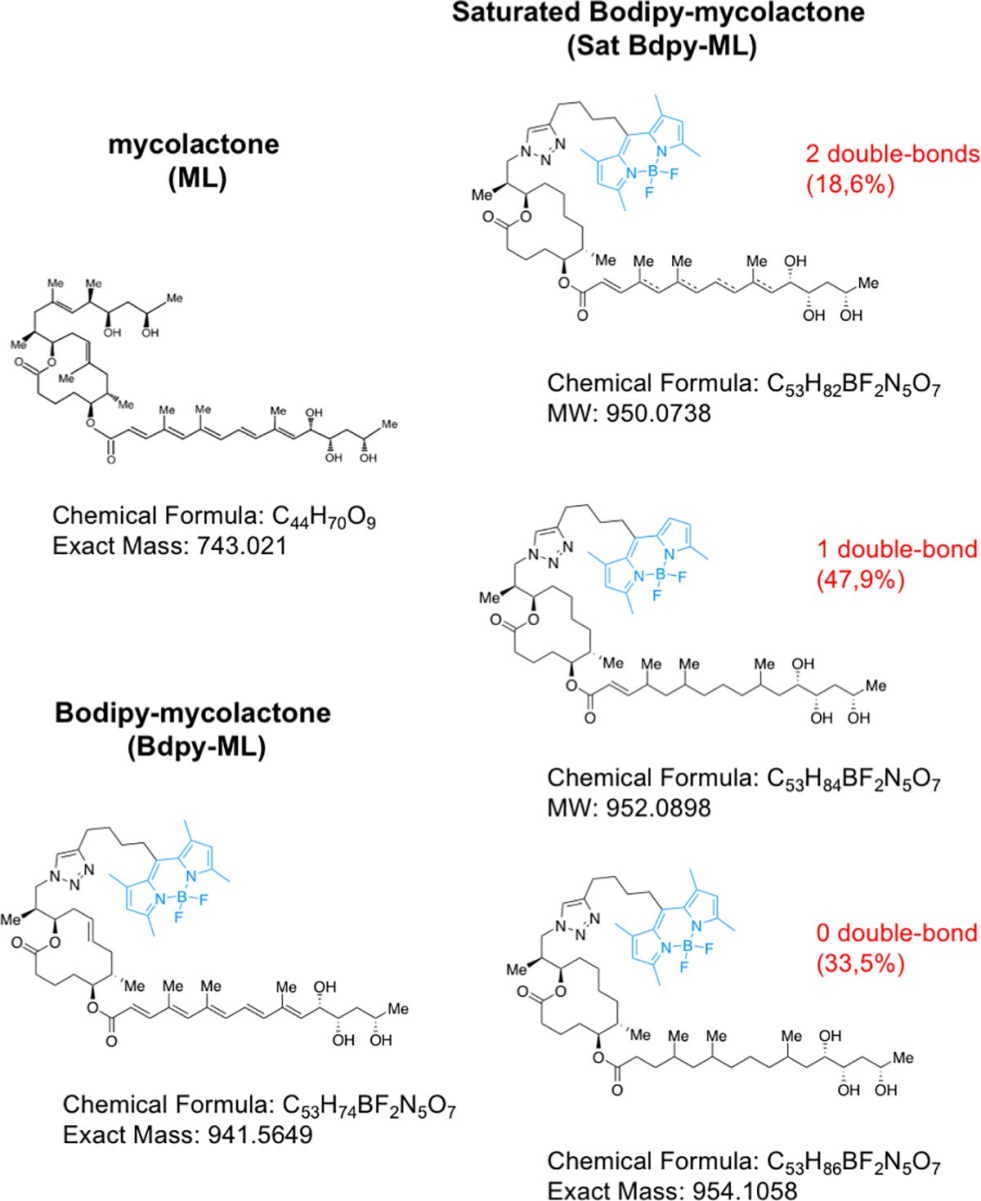
Structures of natural ML and fluorescent derivatives used in this study. Natural ML is constituted of a lactone ring on which are appended two polyketide chains. In Bdpy-ML, the shortest polyketide chain was substituted with a bodipy fluorophore. Saturation of Bdpy-ML’s resulted in Sat-Bdpy-ML, a mixture of compounds presenting two, one or no double bounds within the longest polyketide chain (dotted lines indicate the putative positions of the double bonds).

A green fluorescent derivative of ML was generated by grafting a Bodipy fluorophore onto its smallest polyketide chain (Bdpy-ML, Fig 1), thus preserving the biologically active region of ML *e*.*g*. lactone ring and longest polyketide chain [24,25]. Bdpy-ML retained ML’s ability to block Sec61-mediated production of cytokines and induction of toxicity in cellular assays, albeit with 500-fold less potency (S2 Fig). Bdpy-ML was thus used as a surrogate to visualize diffusion of ML *in vivo*. To see if ML binding to Sec61 interfered with its retention in tissues, we compared the diffusion of Bdpy-ML to that of an isosteric and biologically inert saturated derivative (Sat-Bdpy-ML, Fig 1 and S2 Fig). ML derivatives were injected intravenously in a transgenic zebrafish reporter strain Tg(*Kdrl*:*Ras-mCherry)*. In this strain, expression of the mCherry protein is controlled by the promoter of the VEGF receptor Kdrl, thus labeling endothelial cell membranes in red and allowing to visualize the separation between endothelium and vessel lumen [42]. The amount of injected Bdpy-ML and Sat-Bdpy-ML (7.5 ng/larva) was determined in pilot experiments as optimal for fluorescence acquisition after 24h without induction of adverse effects.

**Fig 2:**
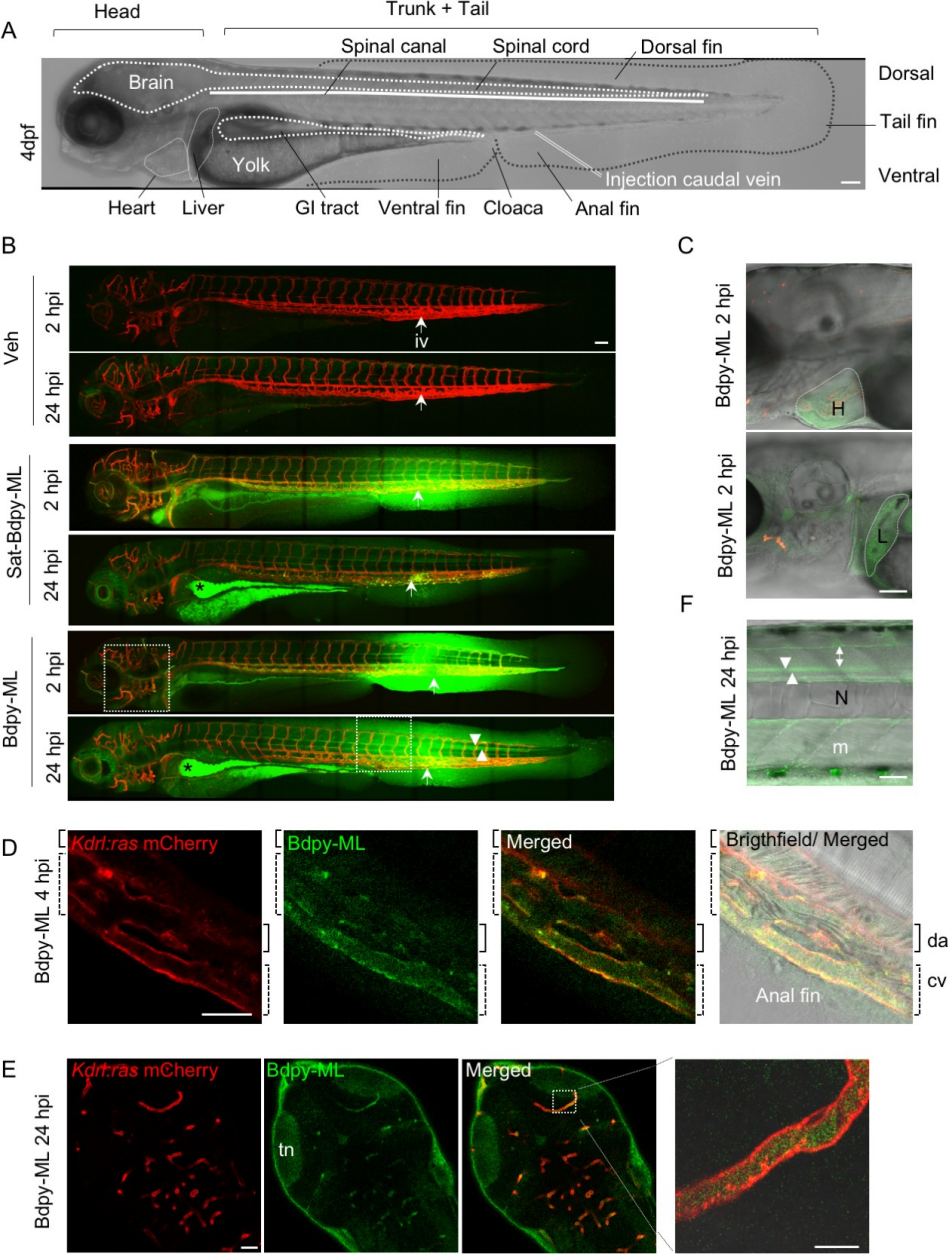
*In vivo* diffusion of Bdpy-ML in zebrafish larva. (A) Brightfield lateral view of a larva at 4 dpf with main anatomical structures highlighted. GI, Gastro-intestinal. (B) lateral views of 4dpf zebrafish *Kdrl*:*Ras mCherry* larvae, 2 and 24 hours post injection (hpi) in the caudal vein with vehicle (Veh), Sat-Bdpy-ML or Bdpy-ML. Images are maximal z projections of confocal tile scan images acquired on same larvae over time; mCherry fluorescence shown in red, Bdpy in green. Arrowheads indicate the spinal canal, arrows indicate the site of intravenous injection (iv), stars show accumulation in the GI tract at 24h. Areas delimited by dotted lines in (B) are shown at higher magnification in (C, 2 hpi) and (F, 24 hpi). Scale Bar 100 μm. (C) Enlarged views (with transmitted light image overlaid on fluorescence) of (B) showing accumulation of Bdpy-ML in heart and liver 2 hpi. (D) Detail of the vasculature in the ventral region of the tail showing longitudinal section of blood vessels and colocalization of Bdpy-ML with mCherry in endothelial cells of the caudal vein 4 hpi. N: notochord, cv (dotted brackets): caudal vein, da (brackets): dorsal aorta. (E) Dorsal view of the head with sections of blood vessels showing blood circulating Bdpy-ML and diffusion into the brain with accumulation in the tectal neuropil (tn) at 24 hpi. Right panel is an enlarged view a blood vessel in longitudinal section. (F) Enlarged view of the trunk in (B) showing preferential accumulation of Bdpy-ML in muscles (m), spinal canal (arrowheads) and spinal cord (double arrow) 24 hpi. N: notochord. (A, B, C, F): lateral views, dorsal top, anterior left; (D) lateral view, dorsal top right, anterior top left; (E) dorsal view, anterior down left. Scale bars (C-E) 40 μm. All images are representative of at least 10 larvae from 3 independent experiments. Images in (C-F) correspond to one focal plane (z = 2 μm).

**Fig 3:**
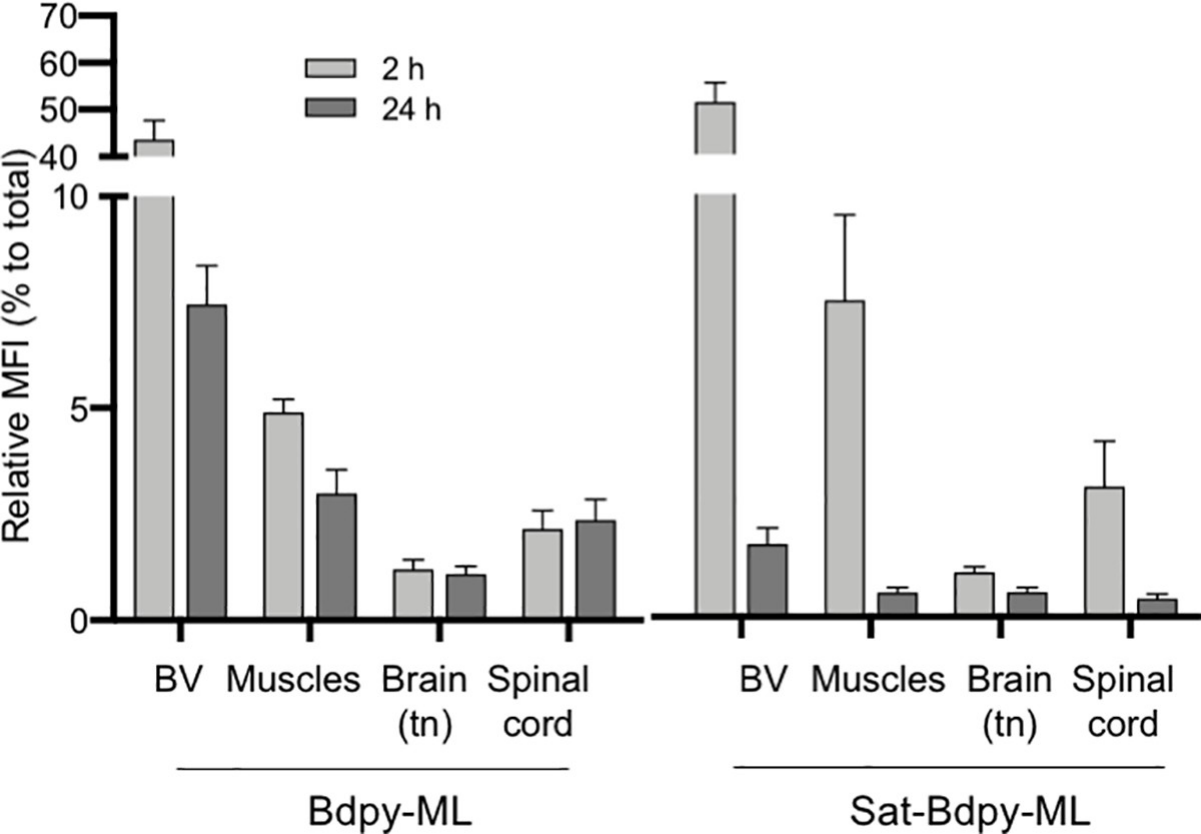
Quantification of Bdpy-ML diffusion in zebrafish larva. Relative fluorescence (to total fluorescent signal) of Bdpy-ML (left) and Sat-Bdpy-ML (right) in blood vessels (BV), muscles, brain and spinal cord, 2 h (light grey) and 24 hpi (dark grey). Data are mean percentages +/- SD. Fluorescence signals were measured on >6 larvae per group with acquisitions in 2 different focal planes (z = 2 μm) for each region, using the same settings.

Tg*(kdrl*:*Ras-mCherry)* larvae were injected in the caudal vein with Bdpy-ML or Sat-Bdpy-ML, or an equivalent volume of vehicle at 4dpf (Fig 2A), and fluorescence signals were acquired by live confocal imaging 2 and 24h post-injection (Fig 2). After 2h, most of the Bdpy-ML and Sat-Bdpy-ML fluorescence signals were detected in the tissues surrounding the site of injection (muscles; dorsal and anal fins) (Fig 2B). Diffusion of both fluorescent compounds was also observed in distant vessels (S3 Fig) and highly perfused organs such as heart and liver (Fig 2B and 2C), although to a lesser degree in the case of Bdpy-ML. This latter observation might be explained by the increased proportion of tetrahedral (Fsp^3^) carbons in Sat-Bdpy-ML, compared to Bdpy-ML (Fsp^3^ Sat-Bdpy-ML = 0.721; Fsp^3^ Bdpy-ML = 0.528, where Fsp^3^ = number of sp^3^ hybridized carbons/ total carbons), which increase lipophilicity thus facilitating diffusion into organs [43]. Notably, Bdpy-ML co-localized with *Kdrl*:*Ras-mCherry* signals in endothelial cells lining blood vessels after 4h (Fig 2D) suggesting that ML extravasates in a transcellular manner or through endocytosis by the highly endocytic endothelial cells of the caudal vein plexus [44]. After 24h, a strong accumulation of Bdpy-ML and Sat-Bdpy-ML was detected in the lipid-rich yolk and in gastro-intestinal (GI) tract (Fig 2B), suggesting elimination via this route. Notably, a significant part of intravenously-delivered Bdpy-ML remained in blood vessels after 24h (Figs 2E and 3), indicating that ML is partly retained in the bloodstream. While the fluorescent signal of Sat-Bdpy-ML had dramatically decreased everywhere else, a strong Bdpy-ML signal was still observed around the injection site after 24h (Fig 2B) and significant Bdpy-ML signal had persisted in muscles (Figs 2F and 3). Compared to Sat-Bdpy-ML, the signal of Bdpy-ML was also higher in the spinal cord and in the spinal canal (Figs 2F and 3) as well as in the brain, particularly in the neuropil region of the optic tectum (Figs 2E and 3). In all, our investigations in the zebrafish model suggested that ML diffuses rapidly from the bloodstream to body tissues, while being partially excreted. Moreover, detection of Bdpy-ML in brain and spinal cord also suggested that ML can cross the BBB.

### Intravenously-delivered ML reaches microglia

**Fig 4:**
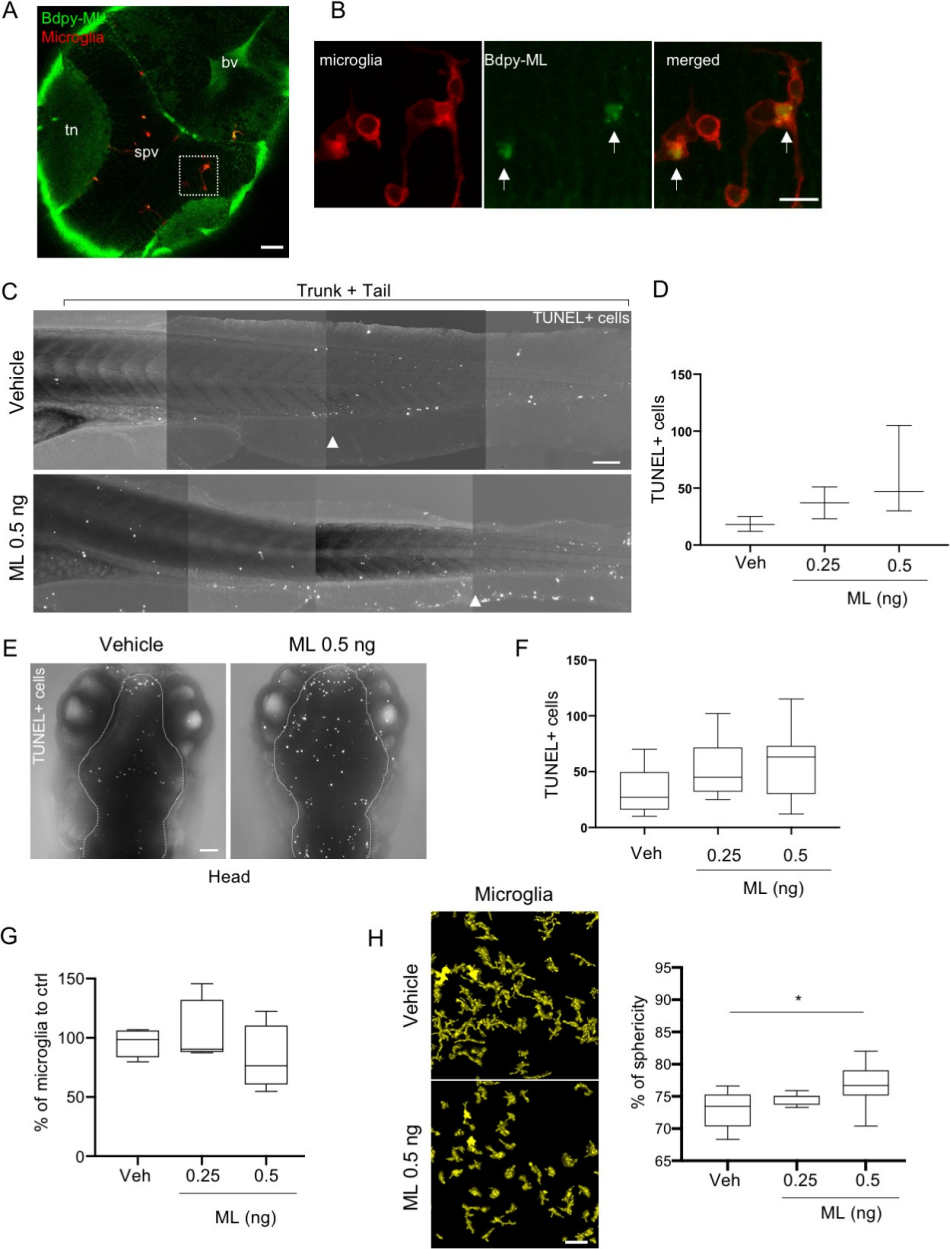
Intravenously-delivered ML gains access to and impact microglia behaviour. (A) Confocal imaging of the brain region of a *mfap4*:*mCherry-F* larva (dorsal view) 24 hpi with Bdpy-ML. Macrophages in red, Bdpy-ML in green. White square indicates enlarged view in (B). tn: tectal neuropil, spv: stratum periventricular, bv: brain ventricles. Scale bar 40 μm. (B) Enlarged view of spv region showing accumulation of Bdpy-ML in microglia (arrows). Images are projections of 4 z stacks (z = 2 μm) scale bar 10 μm. (C) TUNEL labeling of the trunk and tail region of fixed larvae (anterior left, dorsal up), 24 hpi with vehicle or 0.5 ng ML. Arrowheads indicate the site of injection. Scale bar 100 μm. (D) Quantification of TUNEL+ cells in the trunk and tail region. Data are mean +/- SD per larva, 3 larvae per group. (E) TUNEL labeling of brain region (delimited by dotted line) of fixed larvae (dorsal view), 24 hpi with vehicle or 0.5 ng of ML. Images are maximum intensities projection of stacks. Scale bar 40 μm. (F) Quantification of TUNEL+ cells in the brain. Mean +/- SD per larva, > 7 larvae per group. (G) Microglia number, relative to vehicle-injected control. Data are mean percentages to ctrl +/- SD per larva, 3 independent experiments, > 7 larvae per group. (H) Images are 3D reconstructions of microglia (from live imaging) in spv region of vehicle- and ML-injected (0,5 ng) larvae. Scale bar 40 μm. Graph shows mean percentages of sphericity +/- SD of brain microglia in larvae injected with increasing doses of ML (0.25, 0.5 and 1 ng) or corresponding volume of vehicle (veh), as determined with the HK-Means plugin of Icy software. Data are from 3 independent experiments, > 7 larvae per group. Mann-Whitney *P<0.05.

In precedent *in vitro* studies, we had observed that non-cytotoxic doses of ML prevented the pro-inflammatory polarization of microglia and reduced their cytokine production upon activation [1]. To see if intravenously-delivered ML could reach microglia *in vivo*, we injected Bdpy-ML in the bloodstream of 4dpf *mfap4*:*mCherry-F* zebrafish larvae harbouring red fluorescent macrophages and microglia. Consistent with our data shown in Fig 2, upon bloodstream injection, most of the Bdpy-ML reaching the brain 24 hpi localized in the neuropil region of the optic tectum as well as in the brain ventricles (Fig 4A). In addition, we could detect a weak yet significant Bdpy-ML signal within microglia in the stratum periventricular (spv) in the midbrain (Fig 4A and 4B).

Having shown that Bdpy-ML has a broad but heterogenous biodistribution *in vivo*, we next sought to characterize the toxicity of natural ML at the whole-organism level. We injected increasing doses of bacteria-derived ML in the bloodstream of 4dpf *mfap4*:*mCherry-F* larvae, and monitored the effect of ML administration on the entire larvae by evaluating the number of dying cells in the trunk and caudal region, as well as in the brain, and the microglia behaviour 24 hpi. ML doses that could be administered via this route without inducing toxicity at 24 hpi in 4dpf zebrafish larvae were in the 0.25–0.5 ng range (e.g. 0.25–0.5 mg/kg). Of note, these doses were well tolerated and protective in mouse models of skin inflammation or inflammatory pain [12]. In this concentration range, the number of TUNEL+ dying cells in trunk, tail and brain was not significantly modified by ML at 24 hpi (Fig 4C–4F), although a global tendency to an increase could be observed in ML-injected larvae compared to control larvae. In addition, using these doses, we didn’t observe any impact on development, *e*.*g*. inflation of the swim bladder (a specialized air-filled organ that regulates zebrafish buoyancy) was comparable in control and ML-injected larvae 24 hpi. By contrast, swim bladder inflation was impaired and pericardial edema was observed in 90% of the larvae injected with 1 ng ML as well as massive TUNEL labeling (S4 Fig), thus defining 0.5 ng ML as the maximum safe dose to be used in zebrafish larvae at this developmental stage. We cannot exclude that toxicity may develop at later time points in larvae injected with doses up to 0.5 ng. However, we could not image larvae beyond 24 hpi for ethical reasons.

ML injected intravenously at up to 0.5 ng had no significant effect on the total number of microglia (Fig 4G). However, we observed a significant increase in the sphericity of microglia in larvae intravenously injected with 0.5 ng ML (Fig 4H). The same phenotype was observed for tail macrophages (S5 Fig). Macrophage/ microglia morphology being closely related to their function [45] such changes may indicate a modification of their activation status. Alternatively, since cell rounding of adherent cells has been described *in vitro* as an event preceding cell death [46], they may reflect the early stages of an on-going stress response. Yet, this observation suggested that in zebrafish intravenously injected with non-lethal doses of ML, the amount of ML gaining access to microglia is sufficient to alter their biology, and support the fact that ML is able to cross the BBB.

### ML is able to cross an *in vitro* model of human blood brain barrier

**Fig 5:**
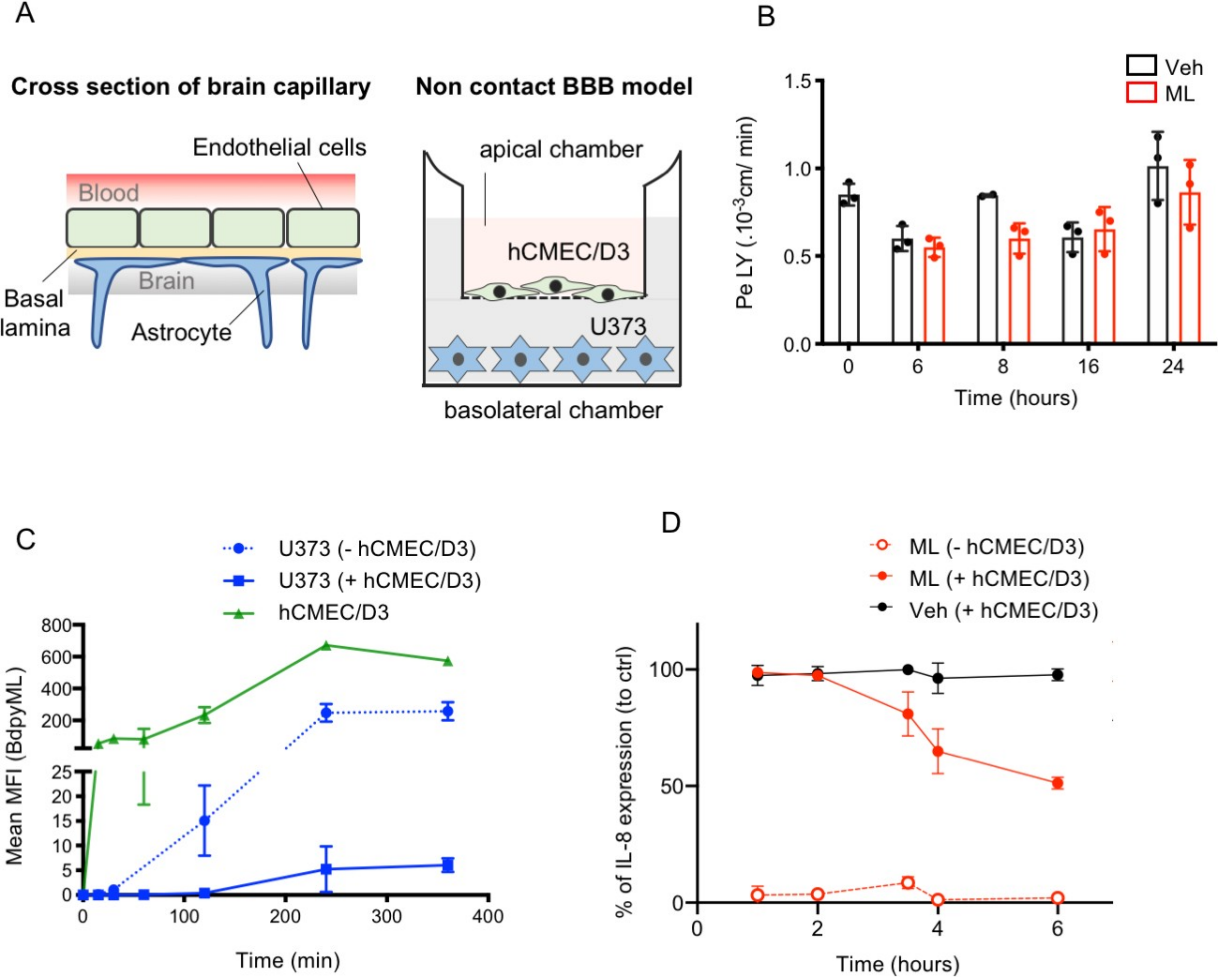
ML crosses an *in cellulo* model of human blood brain barrier. (A) left panel illustrates the basic structural organization of the BBB, right panel shows the *in vitro* transwell, non-contact BBB model used in this study. The human brain capillary endothelial hCMEC/D3 cells are seeded on the apical side of the insert and astrocytes U373 on the bottom of the baso-lateral compartment. Apical and basolateral compartment respectively mimic blood and brain compartments. (B) Permeability to LY as expressed in cm/min, after 6 to 24 h exposure to 1 μg of ML (red bars) or vehicle (DMSO, black bars). Data are means of 3 replicates +/- SD, and are representative of 3 independent experiments. Two-way Anova, no statistically significant difference between ML-treated and vehicle controls. (C) Incorporation of fluorescence across time (as detected by flow cytometry) in hCMEC/D3 (green line) and U373 cells (blue lines) following addition of 2.5 μg of Bdpy-ML on apical compartment in presence (plain line) or not (dotted line) of hCMEC. Data are mean MFI +/- SD of triplicates. (D) Production of IL-8 (expressed in percentage to control) by LPS-activated U373 cells after up to 6 hours of crossing of vehicle (Veh, black curve) and ML (red curves) on filter without hCMEC/D3 (dotted line) or with hCMEC/D3 (plain line). Data are mean percentages of 2 independent experiments in triplicates.

The capacity of Bdpy-ML and natural ML to cross the BBB was further examined with a well-established model of human BBB [37]. This model consists in a monolayer of the human cerebral microvascular endothelial cell line (hCMEC/D3) grown on a porous transwell device, and a monolayer of the human astrocyte cell line U373 (ECACC #08061901) grown in a basolateral chamber (Fig 5A). In this system, the apical and basolateral compartments mimic the blood and brain compartments, respectively. After 6 days of growth, hCMEC/D3 cells formed tight junctions (S6A Fig) restricting paracellular permeability to Lucifer yellow (LY) (Fig 5B). We next tested the capacity of Bdpy-ML added onto the hCMEC/D3 monolayer to reach U373 cells in the basolateral chamber. To this end, hCMEC/D3 and U373 cells were detached and analyzed for Bdpy-ML fluorescence by FACS, at different times after addition of Bdpy-ML into the apical chamber. We used a Bdpy-ML concentration of 2.5 μg/ml that did not affect the hCMEC/D3 barrier permeability within the time frame of the experiment (S6B Fig). Acquisition of fluorescence by hCMEC/D3 cells was detected within 15 min of addition of Bdpy-ML and increased over time to reach a plateau after 4h (Fig 5C). Notably, weak yet significant Bdpy-ML signal was also detected in U373 cells after 4h (Fig 5C). In comparison, the fluorescence acquired by U373 cells in the same experimental set-up without hCMEC/D3 monolayer was higher. The percentage of Bdpy-ML crossing the hCMEC/D3 monolayer, estimated by comparing the level of fluorescence in U373 cells in the absence or presence of this monolayer, was 2,7%. These data show that a small but non-negligeable fraction of Bdpy-ML is able to cross the restrictive hCMEC/D3 monolayer. While additional work will be needed to conclude, our data in Fig 5 and S6 Fig suggest that ML has ability to cross endothelial cell monolayers through the transcellular route.

To validate these results with natural ML, we measured the production of IL-8 by stimulated U373 cells, as a surrogate marker of ML-mediated Sec61 inhibition. Here, 1 μg ML was added onto the hCMEC/D3 monolayer for 1 to 6 h, after which the apical chamber was removed and U373 cells were stimulated overnight with LPS. Once removed, the restrictive capacity of hCMEC/D3 monolayers was controlled by permeability to LY assays and immunostaining (Fig 5B and S6A Fig). The concentration of IL-8 was then assessed in U373 culture supernatant, as an indirect measure of ML-mediated Sec61 blockade in these cells [47]. A time-dependent reduction in IL-8 production was observed 3h after addition of ML to the hCMEC/D3 monolayer (Fig 5D), indicating that ML had blocked Sec61-mediated protein translocation in U373 cells. Using a dose-response curve, we were able to estimate that 1.4% of added ML had crossed the barrier after 6h of incubation (S6C Fig). Since treatment of hCMEC/D3 monolayer with 1 μg/ml ML for up to 24h had no significant effect on its permeability to LY compared to vehicle, (Fig 5B), our data in Fig 5 revealed that ML has the capacity to cross human endothelial barriers, including the BBB. Together with our data acquired in zebrafish, these observations support the view that ML can diffuse from the systemic circulation to the body’s tissues, and gain access to the brain.

### Bacterially-produced ML gains access to the brain

**Fig 6:**
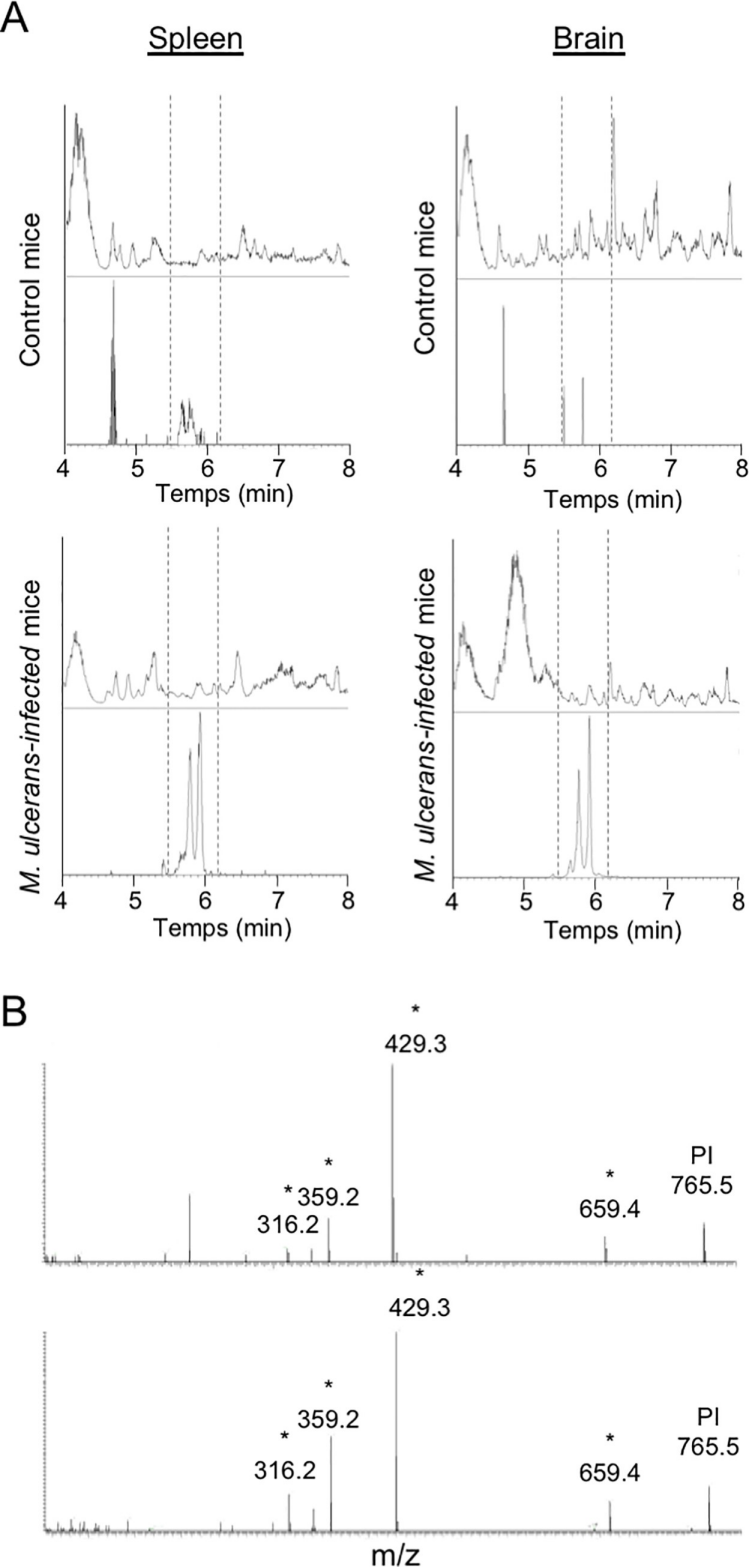
ML is detected in the brain of *M*. *ulcerans* infected mice. (A) UHPLC elution profiles obtained after elution of spleen (left panels) or brain (right panels) lipid extracts from non-infected (Control) or *M*. *ulcerans*-infected mice. Lipid extracts have been prepared from pools of 6 organs. For each UHPLC profile are shown the total ion current (top) and ML’s ion extract [M+Na]+ = 765.4913 (bottom). (B) MS/MS ML’s spectrum showing parental (PI) and fragmentation ions (stars) for pure (top) or ML identified in brain’s lipid extract of *M*. *ulcerans* infected mice.

Previous studies using mice experimentally infected with *M*. *ulcerans* have identified ML in spleen, kidneys and liver but failed to detect the toxin in the brain [8]. We re-examined this result using improved detection techniques and a larger pool of mouse organs. Mice were infected subcutaneously in footpads with a bioluminescent strain of *M*. *ulcerans* allowing real-time monitoring of bacterial growth (S7A Fig)[41,48]. As infection reached its plateau after 11 weeks, mice were deeply anesthetized, perfused with PBS to prevent contamination of organ samples by circulating ML, and the presence of ML was assessed by LC-MS/MS analysis of acetone-soluble lipid extracts prepared from pools of 6 homogenized organs. In accordance with previous findings, ML was detected in mouse spleens (Fig 6A) [8]. In addition, we were able to identify the distinctive mass spectrometric signature of ML (S7B Fig) in brain extracts (Fig 6A and 6B). While mice were anesthetized then intracardiacally perfused with PBS prior to brain harvest, we cannot rule out the possibility that part of the detected ML may derive from blood vessels rather than brain tissues. However, together with the data acquired in the zebrafish and in the BBB models, our detection of intact ML in brain extracts suggests that a fraction of the ML produced by bacteria residing in the skin of infected individuals diffuses into the systemic circulation and crosses the BBB.

## Discussion

Here, we used Bdpy-ML, a fluorescent derivative of ML and the zebrafish larva model to provide a first insight into the spatiotemporal distribution of ML at the whole organism level.

Bdpy-ML is a biologically active surrogate of ML although it showed reduced capacity to block Sec61-mediated production of cytokines and to induce toxicity (500- and 600-fold less active respectively, S2 Fig). Altered biological activity could result from a decreased ability to reach intracellular Sec61 and/or a lower affinity of Bdpy-ML for Sec61. Although less active than ML, preservation of biological activity of Bdpy-ML, together with its cytosolic distribution [24,25] indicate the capacity for Bdpy-ML to reach Sec61 *in vitro*.

Our observation that Sat-Bdpy-ML was inactive in cellular assays of Sec61 inhibition suggested that it was unable to stably bind to Sec61. Sat-Bdpy-ML was cleared from larval zebrafish tissues 24 hpi while Bdpy-ML showed tissue persistence. Since both molecules differed in ability to bind Sec61, this suggests that Bdpy-ML’s interaction with Sec61 may contribute to its persistence *in vivo*.

Our findings revealed a broad and body-wide distribution of ML and support the model of a non-uniform distribution of ML in the body in the context of *M*. *ulcerans* infection, with highest concentrations at the sites of production and excretion. The concentration gradient formed by Bdpy-ML around the site of injection in zebrafish larvae is highly consistent with the pathology of BU lesions, where tissue necrosis is centered on bacterial foci [3]. Accumulation of Bdpy-ML in the GI tract of zebrafish larvae is in line with previous studies using mice injected with radiolabeled ML or experimentally infected with *M*. *ulcerans*. In these studies, detection of ML in liver, kidneys and intestine indeed suggested excretion via urine and feces [8].

Bdpy-ML was found to persist in the bloodstream of injected zebrafish larvae 24 hpi, either in cells or associated to carrier proteins in the plasma. Based on our previous detection of ML in the serum of patients with BU [7] and the recent observation that ML associates with high-and low-density lipoproteins [49], we are privileging the hypothesis that a fraction of bacterially-released ML is retained in the vascular system by plasma proteins, thus preventing elimination of the toxin. It highlights the interest of assays of circulating ML for BU disease monitoring and treatment optimization.

We report for the first time the presence of ML in the brain of *M*. *ulcerans*-infected mice. While we can’t exclude that part of detected ML is located in the endothelial cells of brain vessels, this observation, combined with the detection of Bdpy-ML in the brain of intravenously-injected zebrafish (Fig 2) and ML’s ability to cross the BBB as demonstrated with the *in cellulo* model of human BBB (Fig 5), suggests that part of the ML produced in the skin of infected hosts reaches the central nervous system. In the brain of injected zebrafish larvae, Bdpy-ML accumulated in the neuropil of the optic tectum that contains arborization and projections of neurons. Bdpy-ML was also detected within microglia in the stratum periventricular region. Measures of fluorescence intensities indicated that ML reaches the brain in limited amounts (Fig 3). Injection of ML also triggered morphological changes in microglia further supporting that blood-circulating ML can reach the CNS.

From these observations, we can speculate that in patients with BU, part of circulating ML crosses the BBB to accumulate in the brain. However, it is difficult to estimate in which proportion this might occur. In addition, whether this partial diffusion of ML in the brain of infected hosts is sufficient to impact nervous cell biology and alter Sec61-dependent mechanisms of inflammation and pain transmission is not known and remain to be further explored.

## Supporting information

S1 File Synthesis of fluorescent derivatives of mycolactone and their saturated analogues. (DOCX)

S2 File Permeability to LY assay. Raw data.
(XLS)

S1 Fig Alignment of Danio rerio and Homo sapiens Sec61α protein sequences. Red rectangles indicate putative site of binding for ML [10].
(TIFF)

S2 Fig Compared biological activities of ML, Bdpy-ML and Sat-Bdpy-ML. (A) Dose-dependent suppression of the activation-induced production of IL-2 by Jurkat T cells in the presence of increasing doses of ML (black curve, 0–120 nM), Bdpy-ML (light green curve, 0–15μM) or Sat-Bdpy-ML (dark green curve, 0–15μM). (B) Dose-dependent impact of ML, Bdpy-ML and Sat-Bdpy-ML on Hela cells viability after 48 h of exposure as defined by MTT assay. Means +/- SD of triplicates.
(TIF)

S3 Fig Blood-circulating Bdpy-ML. Dorsal view of the head region of a *kdrl*:*Ras-mCherry* larva (red), 2 h post-injection with Bdpy-ML (green) showing circulation of Bdpy-ML in blood vessels (top panels). Anterior part bottom left. Bottom panels show a zoomed view of blood vessels (white dotted square). Anterior part top left. 1 focal plane, z = 2um. Scale bar 40 μm.
(TIF)

S4 Fig High dose of ML induces massive toxicity. TUNEL labeling of the trunk and tail region of fixed larva (anterior left, dorsal up), 24 hpi with 1 ng ML. Arrowheads indicate the site of injection. Scale bar 100 μm.
(TIFF)

S5 Fig IV-delivered ML impacts macrophages. (A) Z-projection of caudal region of control (top panels) and ML-injected larva (bottom panels) showing microglia in red (left) and their corresponding 3D reconstructions (right). (B) Mean percentage of sphericity of tail macrophages in control ML-injected larvae with increasing doses of ML. Analysis done with the HK-Means plugin of Icy software on 3 larvae per dose, on at least 44 macrophages or 100 microglia per larva. Mann-Whitney *P<0.05.
(TIFF)

S6 Fig Effect of ML on the permeability of a hCMEC/D3 monolayer to LY. (A) Immunostaining of a hCMEC/D3 confluent monolayer showing a continuous staining of adherens junction protein pan-cadherin and tight junction adaptor protein ZO-1 following exposition to vehicle (Veh, top panel) or ML (bottom) during 6h. (B) Permeability of hCMEC/D3 monolayer to LY after 6 h exposure to 2.5 μg of Bdpy-ML or corresponding vehicle. Data are means of 3 replicates +/- SD, and are representatives of 3 independent experiments. (C) Dose-response curve showing production of IL-8 (expressed in percentage to control) by LPS-activated U373, cultured in the same conditions as in BBB assay, and exposed to increasing doses of ML. By reporting the percentage of IL-8 production by activated U373 cells following incubation of vehicle (Veh, black dotted line) or ML (red dotted lines) during 6h on luminal compartment, we were able to estimate the proportion of ML having reached the bottom compartment. Data are means of 2 triplicates.
(TIF)

S7 Fig Mouse infection with bioluminescent M. ulcerans. (A) IVIS monitoring of bioluminescence in control (Ctrl, blue curve) or *M*. *ulcerans* infected mice (Infected, red curve) up to 11 weeks following injection with vehicle or bacterial suspension respectively in the footpads. Mice were sacrificed 11 weeks post-infection. 6 mice per group. (B) Ion extract of purified mycolactone and its isotopic pattern in high resolution MS.
(TIFF)
