# Supplementary material for: Spatiotemporal analysis of mycolactone distribution in vivo reveals partial diffusion in the central nervous system
Source: PLoS Negl Trop Dis. 2020 Dec 2;14(12):e0008878. doi: 10.1371/journal.pntd.0008878 (PMC7710047; doi:10.1371/journal.pntd.0008878)
Supplement: S1 File — (DOCX) [file pntd.0008878.s001.docx]

**Supporting Information**

**Synthesis of fluorescent derivatives of mycolactone and their saturated analogues.**

A first-generation synthesis of fluorescent derivatives of mycolactone has been reported in 2011.^[[1]](#footnote-1)^ We report herein an improved procedure in terms of yields as well as the preparation of new saturated analogues of these fluorescent mycolactones (**Sat-Bdpy-ML**).

**SI-Scheme 1**

To a solution of (S)-2-((2R,7S,8S,E)-8-((tert-butyldimethylsilyl)oxy)-7-methyl-12-oxooxacyclododec-4-en-2-yl)propyl-4-methylbenzenesulfonate **SI-1**^1^ (91 mg, 0.169 mmol) in DMF (1.1 mL) was added sodium azide (20.9 mg, 0.321 mmol). The reaction mixture was warmed up to 75 °C and stirred for 4.5 hours. Then, the mixture was cooled to 0 °C before being diluted with water and diethyl ether. The aqueous layer was washed three times with diethyl ether. The organic layers were combined, washed with brine, dried over anhydrous sodium sulfate, filtered and concentrated to give **SI-2** (71 mg, quantitative yield) as a yellowish oil.

^1^H NMR (300 MHz, CDCl_3_) δ 5.42 (m, 1H), 5.13 (m, 1H), 4.84 (m, 1H), 3.35 (dd, J = 12.2, 5.0 Hz, 1H), 3.30 (m, 1H), 3.10 (dd, J = 12.2, 7.7 Hz, 1H), 2.51 (ddd, J = 11.9, 4.2, 3.4, Hz, 1H), 2.28 (m, 1H), 2.05 (m, 1H), 1.99-1.89 (m, 3H), 1.87-1.83 (m, 2H), 1.76-1.68 (m, 2H), 1.61-1.57 (m, 2H), 0.99 (d, J = 6.5 Hz, 3H), 0.92 (d, J = 6.7 Hz, 3H), 0.86 (s, 9H), 0.01 (s, 6H).

To a 50 mL polypropylene vial containing a solution of **SI-2** (71 mg, 0.173 mmol) in pyridine (5.7 mL) was added HF•pyridine 70% (2.02 mL, 77.9 mmol) at 0 °C. The reaction mixture was warmed up at 40 °C and stirred overnight. The solution was then cooled in an ice bath and a saturated aqueous NaHCO_3_ solution was added until gas evolution stopped. The reaction mixture was diluted with Et_2_O and the layers were separated. The organic layer was washed with brine, dried over anhydrous sodium sulfate, filtered and concentrated to give **SI-3** (51 mg, quantitative yield) as a light yellowish oil.

^1^H NMR (300 MHz, CDCl_3_) δ 5.43 (m, 1H), 5.22 (m, 1H), 4.97 (ddd, J = 11.6, 6.7, 2.5 Hz,1H), 3.46 (m, 1H), 3.37 (dd, J = 12.1, 5.2 Hz, 1H), 3.41 (dd, J = 12.2, 7.5 Hz, 1H), 2.45-2.33 (m, 2H), 2.24-2.16 (m, 2H), 2.00-1.81 (m, 3H), 1.72-1.59 (m, 3H), 1.54-1.42 (m, 2H), 1.03 (d, J = 6.6 Hz, 3H), 0.99 (d, J = 6.4 Hz, 3H).

^13^C NMR (100 MHz, CDCl_3_) δ 174.0, 135.1, 126.1, 73.9, 73.3, 54.0, 38.0, 37.8, 36.8, 36.5, 35.2, 33.0,

19.3, 18.3, 14.4

To a stirred solution of (2E,4E,6E,8E,10E,12S,13S,15S)-12,13,15-tris(tert-Butyldimethylsilyloxy)-4,6,10-trimethylhexadeca-2,4,6,8,10-pentaenoic acid **SI-4**^1^ (224 mg, 0.33 mmol) in benzene (2.79 mL) were added i-Pr_2_NEt (0.204 mL, 1.23 mmol), 2,4,6-trichlorobenzoyl chloride (0.103 mL, 0.66 mmol) and DMAP (188 mg, 1.54 mmol). The resulting mixture was stirred at room temperature for 10 minutes before the addition of **SI-3** (65 mg, 0.22 mmol). The reaction mixture was stirred at room temperature for 15 hours. Then, the reaction was quenched by addition of a saturated aqueous NaHCO_3_ solution and was diluted with Et_2_O. The organic layer was washed with a 1 M aqueous HCl solution, brine, dried over anhydrous sodium sulfate, filtered and concentrated under reduced pressure. The crude product was purified by column chromatography (elution with petroleum ether/EtOAC10:0 to 8:2) to give **SI-5** (215 mg, quantitative yield) as a yellow oil and as a mixture of isomers that was not separated (E-Δ4’-5’/Z-Δ4’-5’ 1:1).

^1^H NMR Z-Δ4’-5’ (400 MHz, CDCl_3_) δ 7.94 (d, J = 15.6 Hz, 1H), 6.48 (dd, J = 14.7, 11.3 Hz, 1H), 6.36 (d, J = 14.7 Hz, 1H), 6.24 (s, 1H), 6.15 (d, J = 11.3 Hz, 1H), 5.87 (d, J = 15.6 Hz, 1H), 5.57 (d, J = 8.3 Hz, 1H), 5.42 (m, 1H), 5.20 (m, 1H), 4.89 (m, 1H), 4.73 (m, 1H), 4.46 (dd, J = 9.1, 3.6 Hz, 1H), 3.90 (m, 1H), 3.62 (m, 1H), 3.37 (m, 1H), 3.12 (m, 1H), 2.48 (m, 1H), 2.33 (m, 1H), 2.19-2.08 (m, 2H), 2.04 (s, 3H), 2.02 (s, 3H), 1.95-1.85 (m, 2H), 1.84 (s, 3H), 1.79-1.56 (m, 8H), 1.13 (d, J = 5.9 Hz, 3H), 1.00 (d, J = 6.8 Hz, 3H), 0.92 (d, J = 6.3 Hz, 3H), 0.87 (s, 9 H), 0.86 (s, 9H), 0.85 (s, 9H), 0.04 (s, 9 H), 0.03 (s, 3H), 0.01 (s, 3H), -0.04 (s, 3H).

^1^H NMR E-Δ4’-5’ (400 MHz, CDCl_3_) δ 7.36 (d, J = 15.5 Hz, 1H), 6.50 (dd, J = 15.0, 11.4 Hz, 1H), 6.36 (d, J = 14.7 Hz, 1H), 6.35 (s, 1H), 6.29 (d, J = 11.4 Hz, 1H), 5.84 (d, J = 15.5 Hz, 1H), 5.54 (d, J = 8.6 Hz, 1H), 5.42 (m, 1H), 5.20 (m, 1H), 4.89 (m, 1H), 4.73 (m, 1H), 4.46 (dd, J = 9.1, 3.6 Hz, 1H), 3.90 (m, 1H), 3.62 (m, 1H), 3.37 (m, 1H), 3.12 (m, 1H), 2.48 (m, 1H), 2.33 (m, 1H), 2.19-2.08 (m, 2H), 2.04 (s, 3H), 2.02 (s, 3H), 1.95-1.85 (m, 2H), 1.84 (s, 3H), 1.79-1.56 (m, 8H), 1.13 (d, J = 5.9 Hz, 3H), 1.00 (d, J = 6.8 Hz, 3H), 0.92 (d, J = 6.3 Hz, 3H), 0.87 (s, 9 H), 0.86 (s, 9H), 0.85 (s, 9H), 0.04 (s, 9 H), 0.03 (s, 3H), 0.01 (s, 3H), -0.04 (s, 3H).

^13^C NMR E-Δ4’-5’ (100 MHz, CDCl_3_) δ 172.8, 166.8, 150.8, 143.7, 139.8, 135.4, 135.2, 134.6, 134.3,

134.1, 132.2, 125.1, 123.7, 116.3, 78.4, 73.6, 72.8, 71.3, 66.0, 54.1, 42.6, 37.9, 37.8, 36.5, 35.4, 34.3,

29.5, 25.9, 25.8, 23.5, 20.4, 19.1, 18.2, 18.1, 17.9, 17.1, 14.4, 14.2, 13.5, -4.2, -4.3, -4.5, -4.6, -4.7.

To a stirred solution of **SI-5** (215 mg, 0.225 mmol) in THF (1.87 mL) was added a 1 M solution of TBAF in THF (2.02 mL, 2.02 mmol). The resulting mixture was stirred at room temperature for 4.5 hours. At the end of the reaction, CaCO_3_ (545 mg), the commercial Dowex® 50WX8-400 hydrogen form resin (1.7 g) and MeOH (3.56 mL) were added and the reaction mixture was stirred for 1 more hour. The crude product was then filtered throught Celite® and concentrated under reduced pressure. The residue was purified by preparative TLC (elution with CH_2_Cl_2_/MeOH 9:1) to give **SI-6** (102 mg, 74%) as a yellowish oil and as a mixture of isomers that was not separated (E-Δ4’-5’/Z-Δ4’-5’ 1:1).

^1^H NMR Z-Δ4’-5’ (400 MHz, Acetone-d6) δ 7.92 (d, J = 15.6 Hz, 1H), 6.66 (dd, J = 14.6, 11.1 Hz, 1H), 6.45 (d, J = 14.6 Hz, 1H), 6.34 (s, 1H), 6.16 (d, J = 11.1 Hz, 1H), 5.94 (d, J = 15.6 Hz, 1H), 5.60 (m, 1H), 5.53 (m, 1H), 5.28 (m, 1H), 4.90 (m, 1H), 4.70 (m, 1H), 4.29 (m, 1H), 4.18 (m, 1H), 4.02 (m, 1H), 3.99 (m, 1H), 3.65 (m, 1H), 3.47 (dd, J = 12.3, 5.2 Hz, 1H), 3.23 (dd, J = 12.3, 7.3 Hz, 1H), 2.86 (br. s, 1H), 2.53 (m, 1H), 2.39 (m, 1H), 2.21 (m, 1H), 2.09 (s, 3H), 2.02 (s, 3H), 1.90 (s, 3H), 1.82 (m, 1H), 1.70-1.65 (m, 5H), 1.55-1.52 (m, 5H), 1.11 (d, J = 6.0 Hz, 3H), 0.99 (d, J = 6.8 Hz, 3H), 0.90 (d, J = 6.2 Hz, 3H).

^13^C NMR Z-Δ4’-5’ (100 MHz, Acetone-d6) δ 173.8, 167.8, 145.3, 144.1, 142.8, 138.3, 136.9, 136.3, 135.8, 135.6, 133.1, 127.5, 126.2, 120.6, 80.1, 76.7, 74.3, 73.3, 68.7, 55.6, 42.8, 39.5, 37.9, 37.0, 36.0, 25.2, 22.0, 21.6, 20.8, 18.6, 18.1, 15.4, 15.3, 14.4.

^1^H NMR E-Δ4’-5’ (400 MHz, Acetone-d6) δ 7.36 (d, J = 15.5 Hz, 1H), 6.64 (dd, J = 15.5, 11.7 Hz, 1H), 6.47 (s, 1H), 6.40 (d, J = 15.5 Hz, 1H), 6.36 (d, J = 11.7 Hz, 1H), 5.89 (d, J = 15.5 Hz, 1H), 5.60 (m, 1H), 5.53 (m, 1H), 5.28 (m, 1H), 4.90 (m, 1H), 4.70 (m, 1H), 4.29 (m, 1H), 4.18 (m, 1H), 4.02 (m, 1H), 3.99 (m, 1H), 3.65 (m, 1H), 3.47 (dd, J = 12.3, 5.2 Hz, 1H), 3.23 (dd, J = 12.3, 7.3 Hz, 1H), 2.86 (br. s, 1H), 2.53 (m, 1H), 2.39 (m, 1H), 2.21 (m, 1H), 2.09 (s, 3H), 2.02 (s, 3H), 1.90 (s, 3H), 1.82 (m, 1H), 1.70-1.65 (m, 5H), 1.55-1.52 (m, 5H), 1.11 (d, J = 6.0 Hz, 3H), 0.99 (d, J = 6.8 Hz, 3H), 0.90 (d, J = 6.2 Hz, 3H).

^13^C NMR E-Δ4’-5’ (100 MHz, Acetone-d6) δ 173.8, 167.8, 152.2, 141.3, 141.0, 138.2, 137.2, 136.9, 136.3, 135.9, 133.1, 127.5, 126.2, 118.4, 80.1, 76.7, 74.3, 73.3, 68.7, 55.6, 42.8, 39.7, 37.9, 36.9, 36.0, 25.2, 22.0, 21.6, 20.8, 18.6, 18.1, 15.4, 15.3, 14.4.

To a solution of **SI-6** (55 mg, 0.0896 mmol) in acetonitrile (2.75 mL) were added 5,5-difluoro-10-(hex-5-yn-1-yl)-1,3,7,9-tetramethyl-5H-dipyrrolo[1,2-c:2',1'-f][1,3,2]diazaborinin-4-ium-5-uide **SI-7** (102 mg, 0.311 mmol), i-Pr_2_NEt (14.8 μL, 0.0896 mmol), CuI (1.71 mg, 0.00896 mmol) and L-ascorbic acid sodium salt (3.55 mg, 0.0179 mmol). The mixture was stirred at room temperature for 2.5 hours. Then, the reaction mixture was evaporated, and the residue was partitioned between EtOAC and water. The organic layer was washed with a saturated aqueous NaHCO3 solution, brine, dried over anhydrous sodium sulfate, filtered and evaporated. The crude residue was purified by column chromatography (elution with CH_2_Cl_2_/MeOH 10:0 to 9:1) to give after evaporation **SI-8** (60 mg, 71%) as a red viscous solid and as a mixture of isomers that was not separated (E-Δ4’-5’/Z-Δ4’-5’ 1:1).

^1^H NMR Z-Δ4’-5’ (400 MHz, Acetone-d6) δ 7.92 (d, J = 15.6 Hz, 1H), 7.74 (s, 1H), 6.64 (dd, J = 15.0, 11.5 Hz, 1H), 6.45 (d, J = 15.5 Hz, 1H), 6.33 (s, 1H), 6.17 (s, 2H), 6.16 (d, J = 11.5 Hz, 1H), 5.94 (d, J = 15.6 Hz, 1H), 5.60 (m, 1H), 5.53 (m, 1H), 5.28 (m, 1H), 5.20 (m, 1H), 4.91 (m, 1H), 4.69 (m, 1H), 4.47 (dd, J =13.7, 5.2 Hz, 1H), 4.28 (m, 1H), 4.22 (m, 1H), 4.15 (m, 1H), 4.05-3.94 (m, 2H), 3.66 (m, 1H), 3.07-3.02 (m, 2H), 2.83-2.77 (m, 3H), 2.52 (m, 1H), 2.45 (s, 6H), 2.43 (s, 6H), 2.35-2.30 (m, 2H), 2.25-2.19 (m, 3H), 2.09 (s, 3H), 2.03 (s, 3H), 1.91 (s, 3H), 1.83 (m, 1H), 1.76-1.66 (m, 8H), 1.54-1.51 (m, 2H), 1.12 (d, J = 6.1 Hz, 3H), 0.91 (d, J = 6.2 Hz, 3H), 0.85 (d, J = 6.9 Hz, 3H).

^13^C NMR Z-Δ4’-5’ (100 MHz, Acetone-d6) δ 174.0, 167.8, 155.3, 148.7, 145.4, 144.1, 142.8, 138.3, 137.0, 136.3, 135.9, 135.8, 135.7, 134.2, 133.1, 127.3, 126.1, 123.8, 123.4, 120.6, 80.1, 76.8, 74.6, 73.3, 68.7, 53.6, 42.8, 40.5, 39.6, 38.0, 36.9, 36.0, 33.0, 29.9, 26.9, 25.3, 22.0, 21.7, 20.8, 18.6, 18.1, 17.4, 15.4, 15.3, 14.4.

^1^H NMR E-Δ4’-5’ (400 MHz, Acetone-d6) δ 7.74 (s, 1H), 7.37 (d, J = 15.4 Hz, 1H), 6.64 (dd, J = 14.8, 11.2 Hz, 1H), 6.46 (s, 1H), 6.40 (d, J = 14.8 Hz, 1H), 6.36 (d, J = 11.2 Hz, 1H), 6.17 (s, 2H), 5.89 (d, J = 15.5 Hz, 1H), 5.60 (m, 1H), 5.53 (m, 1H), 5.28 (m, 1H), 5.20 (m, 1H), 4.91 (m, 1H), 4.69 (m, 1H), 4.47 (dd, J = 13.7, 5.2 Hz, 1H), 4.28 (m, 1H), 4.22 (m, 1H), 4.15 (m, 1H), 4.05-3.94 (m, 2H), 3.66 (m, 1H), 3.07-3.02 (m, 2H), 2.83-2.77 (m, 3H), 2.52 (m, 1H), 2.45 (s, 6H), 2.43 (s, 6H), 2.35-2.30 (m, 2H), 2.25-2.19 (m, 3H), 2.09 (s, 3H), 2.03 (s, 3H), 1.91 (s, 3H), 1.83 (m, 1H), 1.76-1.66 (m, 8H), 1.54-1.51 (m, 2H), 1.12 (d, J = 6.1 Hz, 3H), 0.91 (d, J = 6.2 Hz, 3H), 0.85 (d, J = 6.9 Hz, 3H).

^13^C NMR E-Δ4’-5’ (100 MHz, Acetone-d6) δ 174.0, 167.8, 155.3, 152.2, 148.5, 141.4, 140.9, 138.3, 137.2 137.0, 136.3, 135.9, 135,8, 134.2, 133.1, 127.3, 126.1, 123.8, 123.4, 118.3, 80.1, 76.8, 74.6, 73.3, 68.7, 53.6, 42.8, 40.5, 39.6, 38.0, 36.9, 36.0, 33.0, 29.9, 26.9, 25.3, 22.0, 21.6, 20.8, 18.6, 18.1, 17.4, 15.4, 15.3, 14.4.

To a solution of **SI-8** (5 mg, 5.78 μmol) in EtOH (4 mL) was added a spatula tip of 10% Pd/C. The mixture was stirred under an atmosphere of H2 at room temperature for 15 hours. The reaction mixture was then filtrated over Celite® and evaporated to give **Bdpy-ML** (5.5 mg, quantitative yield) as a mixture of isomers (fully saturated product: 33.5%, mono-alkene product: 47.9%, di-alkene product: 18.6%) that was not separated, as a red solid.

HRMS calculated for **Sat-Bdpy-ML-1**, C_53_H_86_BF_2_N_5_O_7_ 952.6625; found 952.6570 [M-H].

HRMS calculated for **Sat-Bdpy-ML-2**, C_53_H_84_BF_2_N_5_O_7_ 950.6468; found 950.6488 [M-H].

HRMS calculated for **Sat-Bdpy-ML-3**, C_53_H_82_BF_2_N_5_O_7_ 948.6312; found 948.6318 [M-H].

1. Chany, A.-C.; Casarotto, V.; Schmitt, M.; Tarnus, C.; Guenin-Macé, L.; Demangel, C.; Mirguet, O.; Eustache, J.; Blanchard, N., A Diverted Total Synthesis of Mycolactone Analogues: An Insight into Buruli Ulcer Toxins. *Chem. Eur. J.* **2011,** *17*, 14413-14419. [↑](#footnote-ref-1)
