# Supplementary figures and images for: Spatiotemporal analysis of mycolactone distribution in vivo reveals partial diffusion in the central nervous system
Source: PLoS Negl Trop Dis. 2020 Dec 2;14(12):e0008878. doi: 10.1371/journal.pntd.0008878 (PMC7710047; doi:10.1371/journal.pntd.0008878)

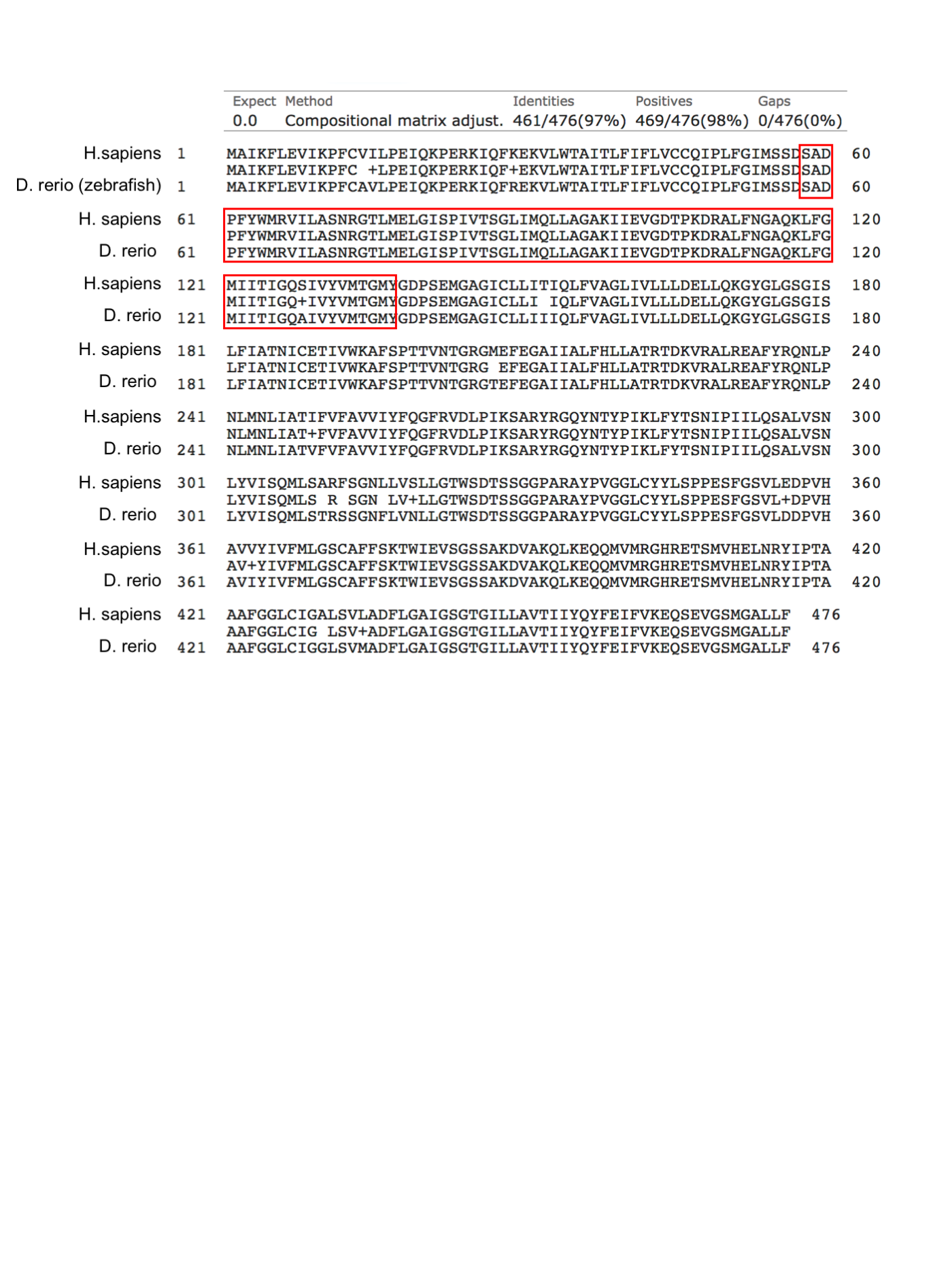

Supplement: S1 Fig — Red rectangles indicate putative site of binding for ML [10]. (TIFF) [file pntd.0008878.s003.tiff]

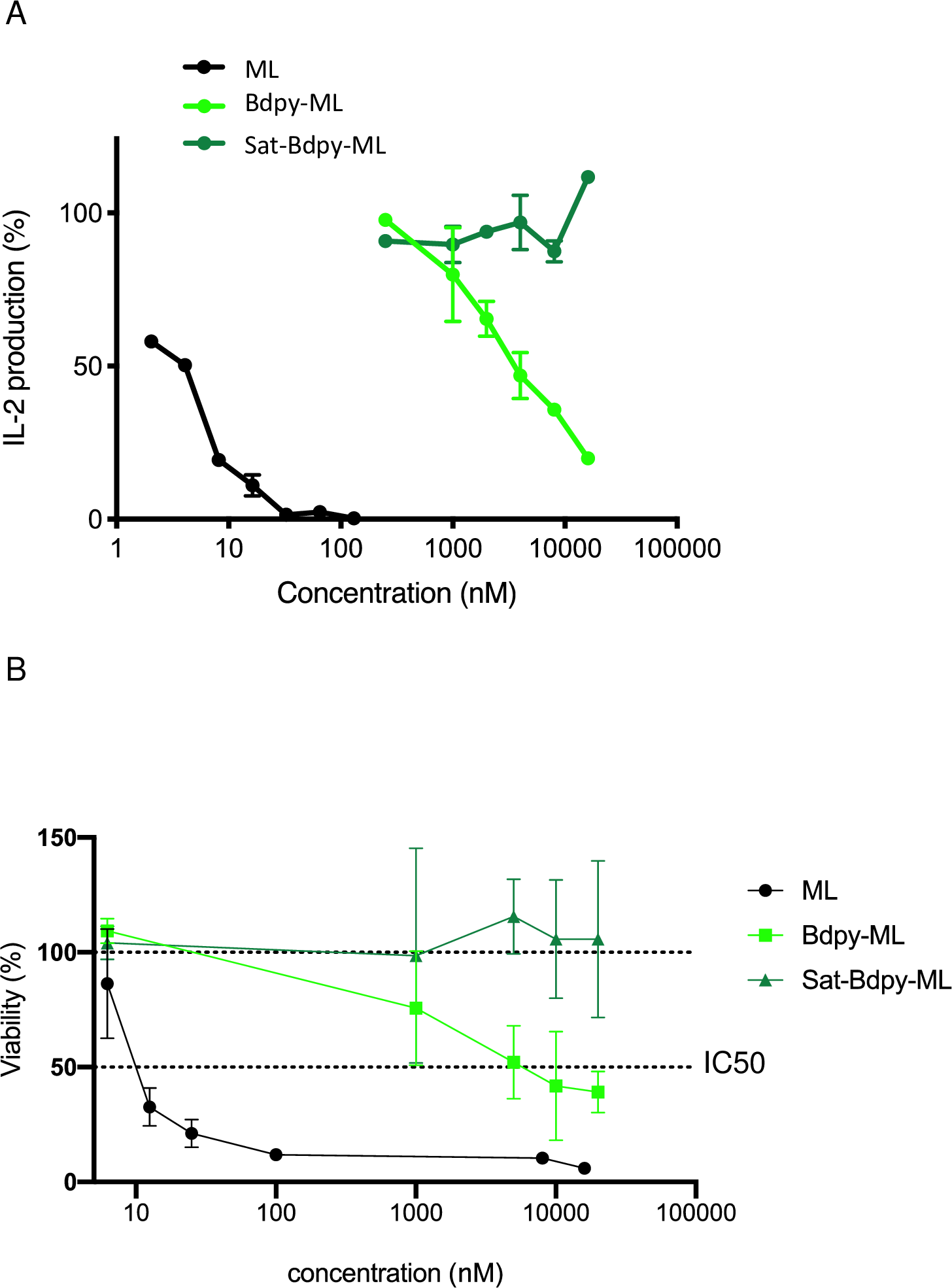

Supplement: S2 Fig — (A) Dose-dependent suppression of the activation-induced production of IL-2 by Jurkat T cells in the presence of increasing doses of ML (black curve, 0–120 nM), Bdpy-ML (light green curve, 0–15μM) or Sat-Bdpy-ML (dark green curve, 0–15μM). (B) Dose-dependent impact of ML, Bdpy-ML and Sat-Bdpy-ML on Hela cells viability after 48 h of exposure as defined by MTT assay. Means +/- SD of triplicates. (TIF) [file pntd.0008878.s004.tif]

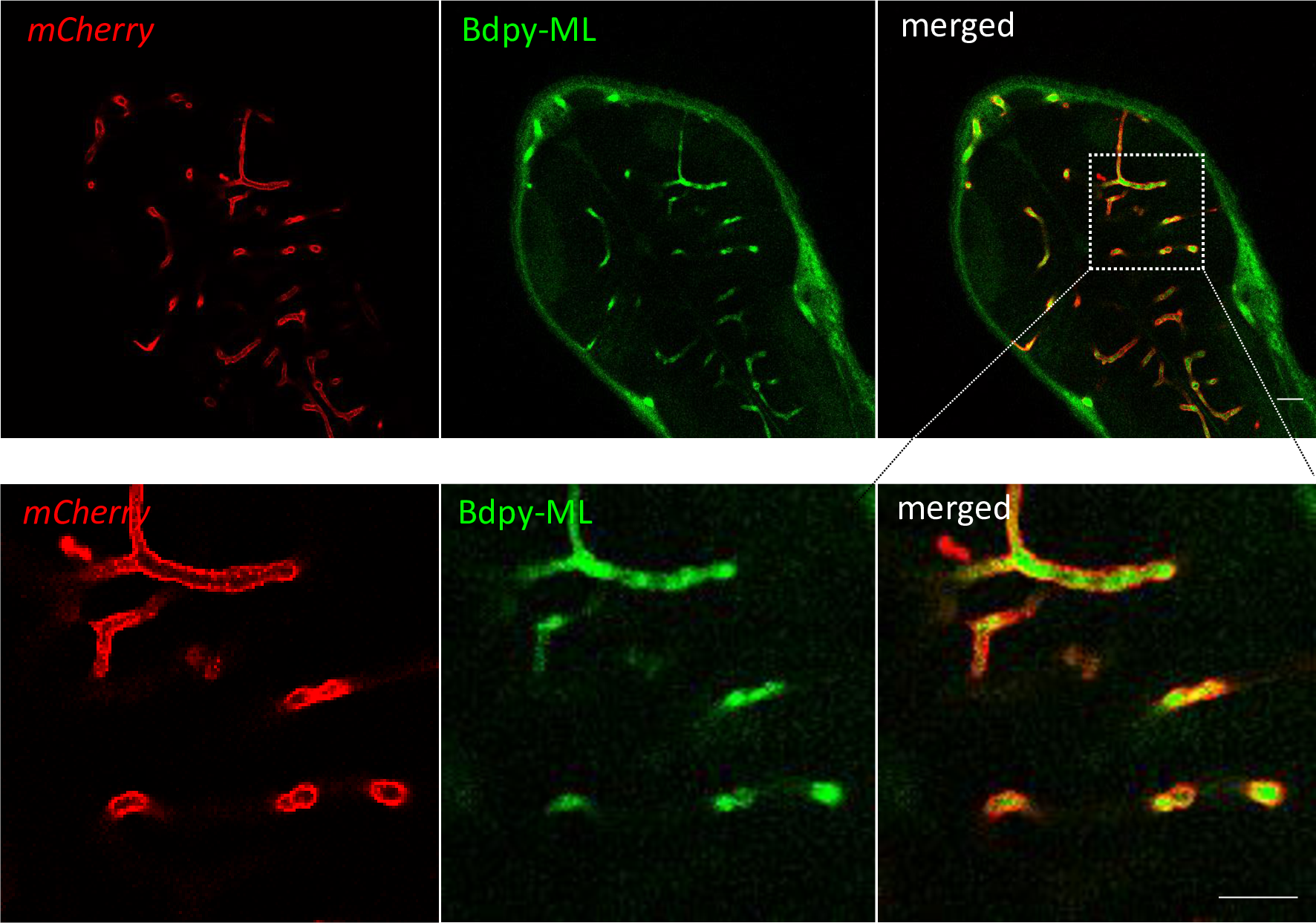

Supplement: S3 Fig — Dorsal view of the head region of a kdrl:Ras-mCherry larva (red), 2 h post-injection with Bdpy-ML (green) showing circulation of Bdpy-ML in blood vessels (top panels). Anterior part bottom left. Bottom panels show a zoomed view of blood vessels (white dotted square). Anterior part top left. 1 focal plane, z = 2um. Scale bar 40 μm. (TIF) [file pntd.0008878.s005.tif]

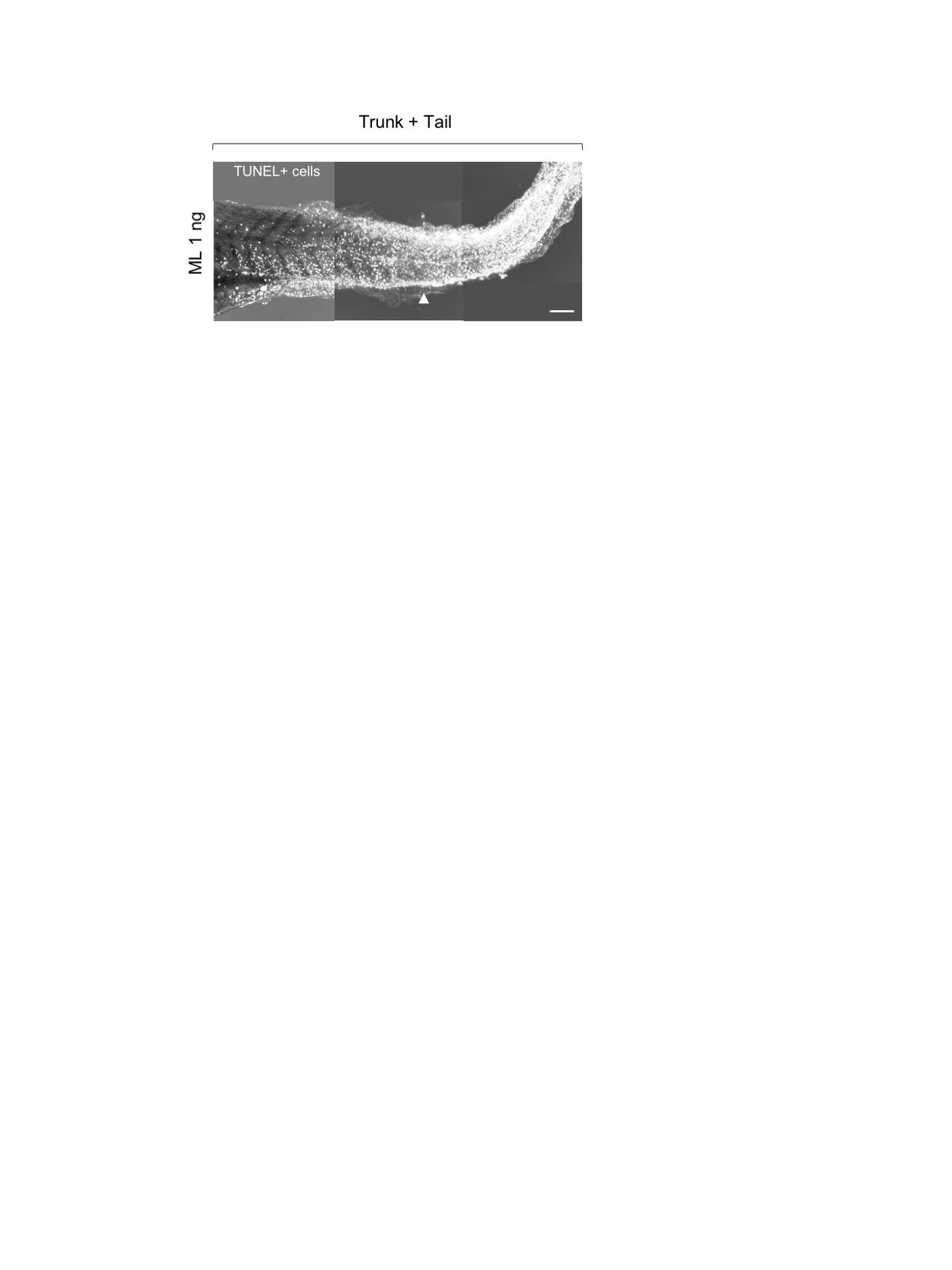

Supplement: S4 Fig — TUNEL labeling of the trunk and tail region of fixed larva (anterior left, dorsal up), 24 hpi with 1 ng ML. Arrowheads indicate the site of injection. Scale bar 100 μm. (TIFF) [file pntd.0008878.s006.tiff]

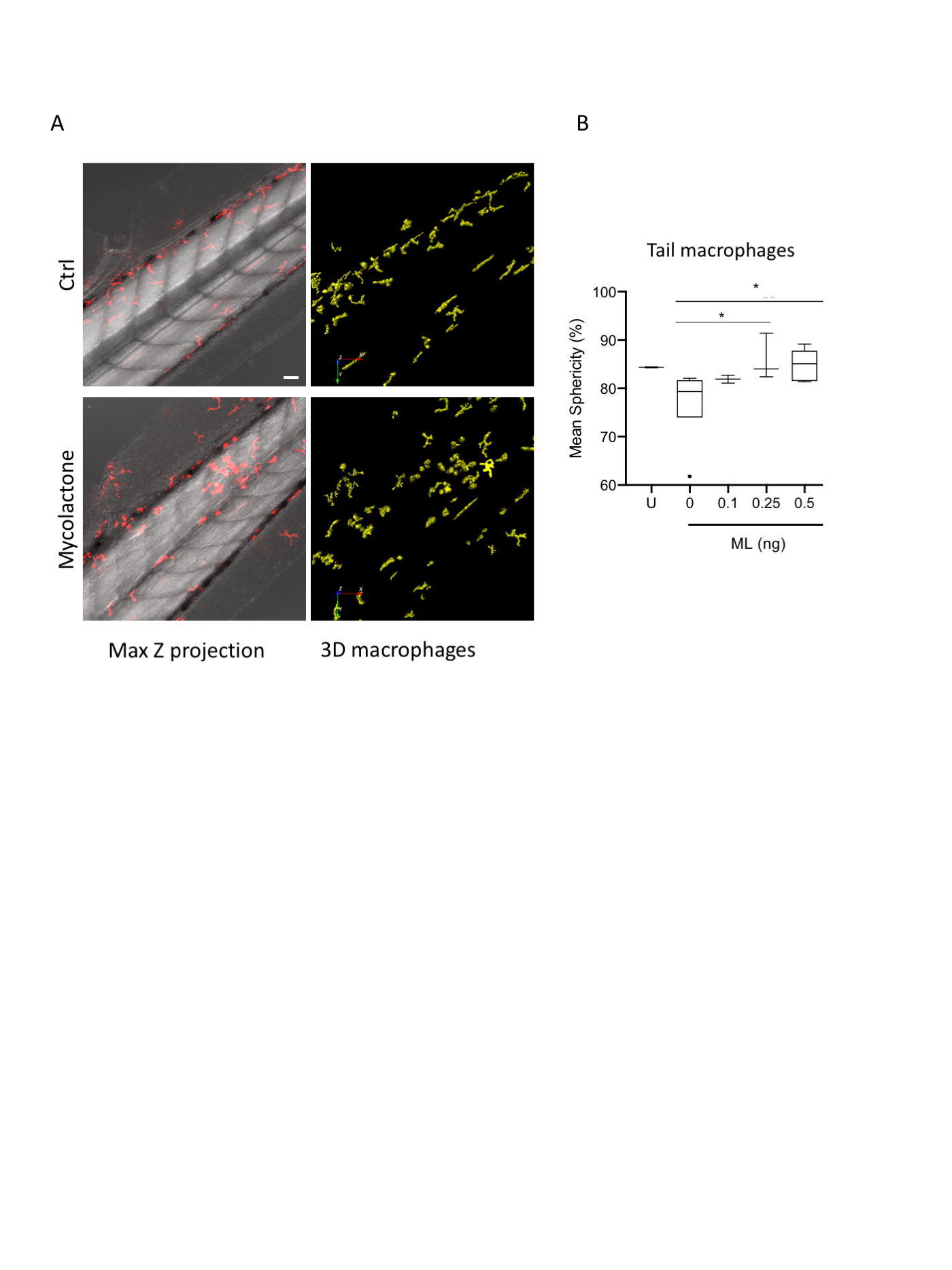

Supplement: S5 Fig — (A) Z-projection of caudal region of control (top panels) and ML-injected larva (bottom panels) showing microglia in red (left) and their corresponding 3D reconstructions (right). (B) Mean percentage of sphericity of tail macrophages in control ML-injected larvae with increasing doses of ML. Analysis done with the HK-Means plugin of Icy software on 3 larvae per dose, on at least 44 macrophages or 100 microglia per larva. Mann-Whitney *P<0.05. (TIFF) [file pntd.0008878.s007.tiff]

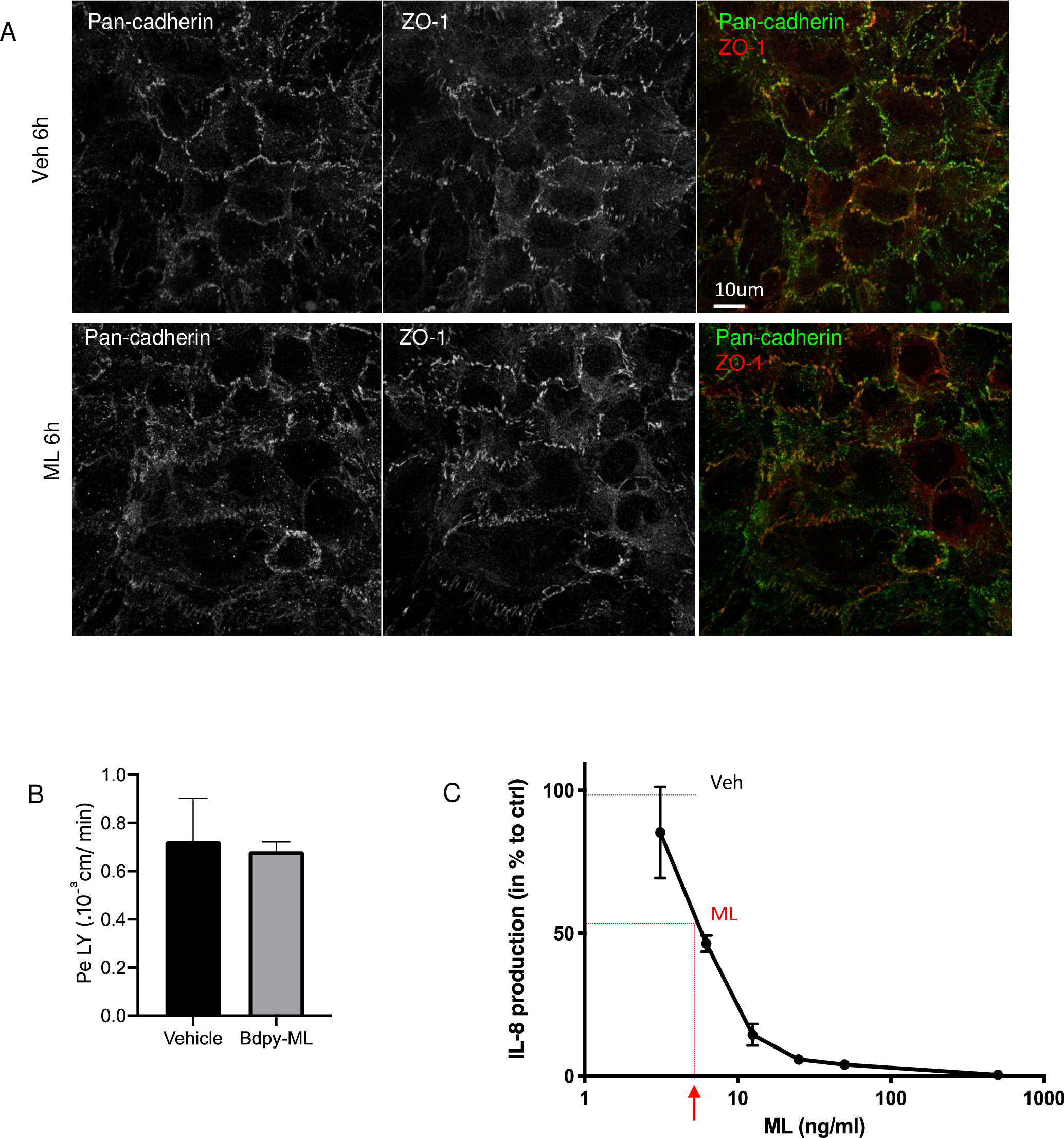

Supplement: S6 Fig — (A) Immunostaining of a hCMEC/D3 confluent monolayer showing a continuous staining of adherens junction protein pan-cadherin and tight junction adaptor protein ZO-1 following exposition to vehicle (Veh, top panel) or ML (bottom) during 6h. (B) Permeability of hCMEC/D3 monolayer to LY after 6 h exposure to 2.5 μg of Bdpy-ML or corresponding vehicle. Data are means of 3 replicates +/- SD, and are representatives of 3 independent experiments. (C) Dose-response curve showing production of IL-8 (expressed in percentage to control) by LPS-activated U373, cultured in the same conditions as in BBB assay, and exposed to increasing doses of ML. By reporting the percentage of IL-8 production by activated U373 cells following incubation of vehicle (Veh, black dotted line) or ML (red dotted lines) during 6h on luminal compartment, we were able to estimate the proportion of ML having reached the bottom compartment. Data are means of 2 triplicates. (TIF) [file pntd.0008878.s008.tif]

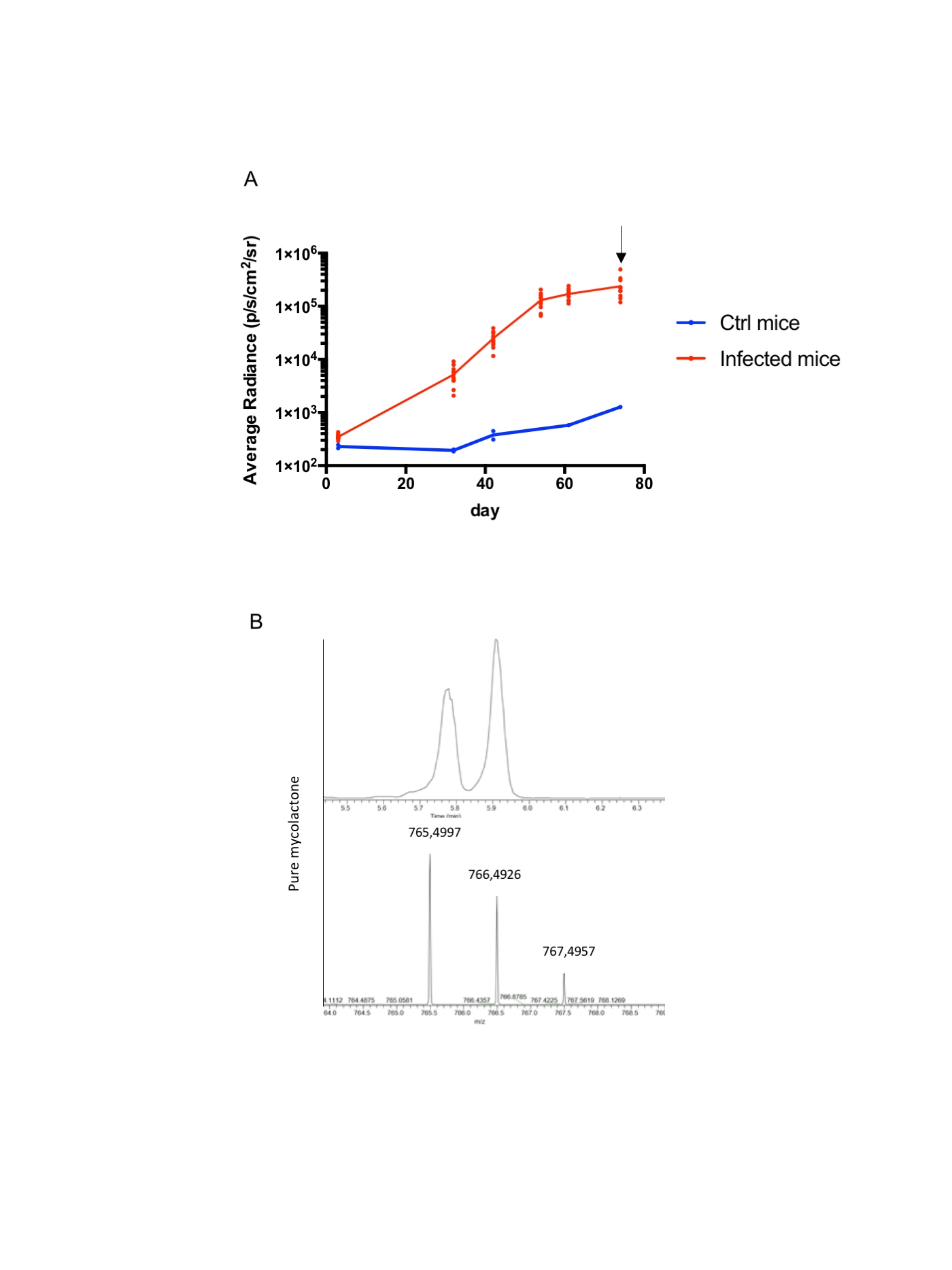

Supplement: S7 Fig — (A) IVIS monitoring of bioluminescence in control (Ctrl, blue curve) or M. ulcerans infected mice (Infected, red curve) up to 11 weeks following injection with vehicle or bacterial suspension respectively in the footpads. Mice were sacrificed 11 weeks post-infection. 6 mice per group. (B) Ion extract of purified mycolactone and its isotopic pattern in high resolution MS. (TIFF) [file pntd.0008878.s009.tiff]
